# Morphology effects on surface chemical properties and lattice defects of Cu/CeO_2_ catalysts applied for low-temperature CO oxidation

**DOI:** 10.1038/s41598-019-48606-2

**Published:** 2019-08-19

**Authors:** Fang Dong, Yu Meng, Weiliang Han, Haijun Zhao, Zhicheng Tang

**Affiliations:** 1 State Key Laboratory for Oxo Synthesis and Selective Oxidation, and National Engineering Research Center for Fine Petrochemical Intermediates, Lanzhou Institute of Chemical Physics, Chinese Academy of Sciences, Lanzhou, 730000 China; 2 Shanxi Key Laboratory of Low metamorphic Coal Clean Ytilization, School of Chemistry and Chemical Engineering, Yulin University, YuLin, 719000 China

**Keywords:** Pollution remediation, Heterogeneous catalysis

## Abstract

Here, we synthesized a series of Cu/CeO_2_ catalysts with different morphology and size, including Cu/CeO_2_ nanospheres (Cu/CeO_2_-S), Cu/CeO_2_ nanoparticles (Cu/CeO_2_-P), Cu/CeO_2_ nanorods (Cu/CeO_2_-R) and flower-like Cu/CeO_2_ microspheres (Cu/CeO_2_-F) to systematically explore the structure-activity relationship in CO oxidation. Crucially, the effect of morphology, crystal size, Ce^4+^/Ce^3+^ species, oxygen vacancies derived from the removal of lattice oxygen (O_latt_) species in CeO_2_ and lattice defect sites on CO activity was revealed through various characterizations. It was clearly discovered that the activity of these catalysts was as follows: Cu/CeO_2_-R > Cu/CeO_2_-P > Cu/CeO_2_-S > Cu/CeO_2_-F, and the Cu/CeO_2_-R catalyst preferentially showed the best catalytic performance with a 90% conversion of CO even at 58 °C, owned the smaller particles size of CeO_2_ and CuO, and exhibited the higher concentration of O_latt_ species and oxygen vacancies. Besides, it is also verified that the Cu/CeO_2_-F sample exhibited the larger CeO_2_ crystal size (17.14 nm), which led to the lower Cu dispersion and CO conversion, even at 121 °C (T_90_). Most importantly, we discovered that the amount of surface lattice defect sites was positively related to the reaction rate of CO. Simultaneously, DFT calculation also demonstrated that the introduced oxygen vacancies in CeO_2_ could accelerate the oxidation of CO by the alteration of CO adsorption energy. Therefore, the morphology, the crystal size, the content of oxygen vacancies, as well as lattice defects of Cu/CeO_2_ catalyst might work together for CO oxidation reaction.

## Introduction

Carbon monoxide (CO) has considered as a toxic air pollutant. Especially, it is harmful to the environment and human health^1–4^. For the emission of CO, a large number of technologies have been developed, including absorption, photocatalytic oxidation, and thermal catalytic oxidation^5–9^. Among, the catalytic oxidation of CO is treated as the most efficient method to eliminate CO, because of the oxidation elimination of CO with the high efficiency and low cost^10–12^. However, developing an effective heterogeneous catalyst to promote CO oxidation at low temperature still remains a great challenge. Therefore, it is highly desirable to develop a series of catalysts with outstanding activity at low temperature, reduce the energy consumption and remove the high concentration CO at low cost.

Recently, a large number of works^13–16^ have verified that transition-metal oxides are promising candidates for CO oxidation. Especially, cerium oxide (CeO_2_) is widely used as an efficient replacement of noble metals in heterogeneous catalytic oxidation reaction^17,18^. The reason is related to its ability to switch between Ce^4+^ and Ce^3+^ oxidation states during the reaction process. Generally, ceria-based catalysts in heterogeneous catalytic field may exist three configurations, including metal/oxide, oxide/metal and {oxide + metal}/oxide^17^. It is worth our attention that the metal/oxide configuration is the most common in industrial application. Accordingly, the catalysts with the metal/oxide configuration have been used to the oxidation reaction of CO as a major air pollutant^19^, such as the synthesis of valuable chemicals derived from the conversion of CO_2_ ^20,21^, the water-gas shift reactions and the reforming of hydrocarbons^22–25^. However, there are some disadvantages when bulk ceria is used as a stable oxide support, which limits the activity of catalysts. For instance, L. Ma *et al*.^26^ reported that Ag/CeO_2_ nanosphere catalyst prepared by a one-step hydrothermal method exhibited much higher catalytic activity in formaldehyde oxidation reaction than normal Ag/CeO_2_ particles prepared by conventional impregnation method, which was attributed to the more surface chemisorbed oxygen on the Ag/CeO_2_ nanosphere catalyst. Besides, L. Qi *et al*.^27^ also reported that the cerium precursors exerted a great influence on the texture and chemical properties of CuO-CeO_2_ catalysts. Therefore, the structure of CeO_2_ as support is vitally significant for improving the activity of ceria-based catalysts.

Besides, it is well-known that copper-based solids are attractive because of their application as efficient catalysts in various redox reactions, including the oxidation of CO and volatile organic compounds^28,29^, alcohol synthesis^30,31^, the water-gas shift reactions and so on^22,32,33^. Numerous works^34–36^ have verified that copper particles size with nanometre range (1 to 100 nm) can greatly promote their catalytic performance, especially in CO and hydrocarbons oxidation reaction. For example, J. Sun *et al*.^37^ discovered that the synergistic effect between the dispersion of copper and ceria species could promote the catalytic performance of CuCeAl catalysts. Recently, W. Wang *et al*.^38^ discovered that the exposed crystal planes of CeO_2_ support and surface copper species are the key factors to determine the catalytic performance of CuCe catalysts. Besides, L. Du *et al*.^39^ also reported that the excessive loadings of copper can strongly affect the dispersion of copper and thus lead to the formation of less active copper species. D. Zhang *et al*.^40^ synthesized a series of Cu-doped CeO_2_ hollow spheres by a simple hydrothermal method, and discovered that the superior catalytic performance for CO oxidation reaction is ascribed to the porous spherical structure, high redox capability and high oxygen vacancy. Accordingly, a series of designed Cu/CeO_2_ catalysts with different morphology can be synthesized and investigated in this work.

Herein, we synthesized a series of CeO_2_ supports with different morphology and size, including CeO_2_ nanoparticles (20 nm), CeO_2_ nanospheres (200 nm), CeO_2_ nanorods (20–40 nm) and flower-like CeO_2_ microspheres (4 µm). After that, copper species (15 wt %) was anchored on these CeO_2_ surface through deposition precipitation method, which made it possible to play the key role in modulating the interaction of Cu species and CeO_2_ derived from the morphology effect. Notably, it was discovered that CuO NPs deposited on nanorods CeO_2_ mainly exposed {110} crystal planes, which was much more reactive than that on nanoparticles CeO_2_ exposed {100} in CO oxidation. More interestingly, it was confirmed that the nanorods Cu/CeO_2_ catalyst was beneficial to the formation of surface oxygen vacancies derived from the removal of lattice oxygen (O_latt_) species in CeO_2_ with the stronger interaction between Cu species and CeO_2_ nanorods, while the flower-like CeO_2_ microspheres was adverse for the formation of oxygen vacancies. Besides, the morphology of CeO_2_ support greatly altered the particles size of CeO_2_ and CuO, which were also considered as one of factors to determine the catalytic performance of Cu/CeO_2_ catalysts.

## Results and Discussion

### Catalytic performance in CO oxidation reaction

**Figure 1:**
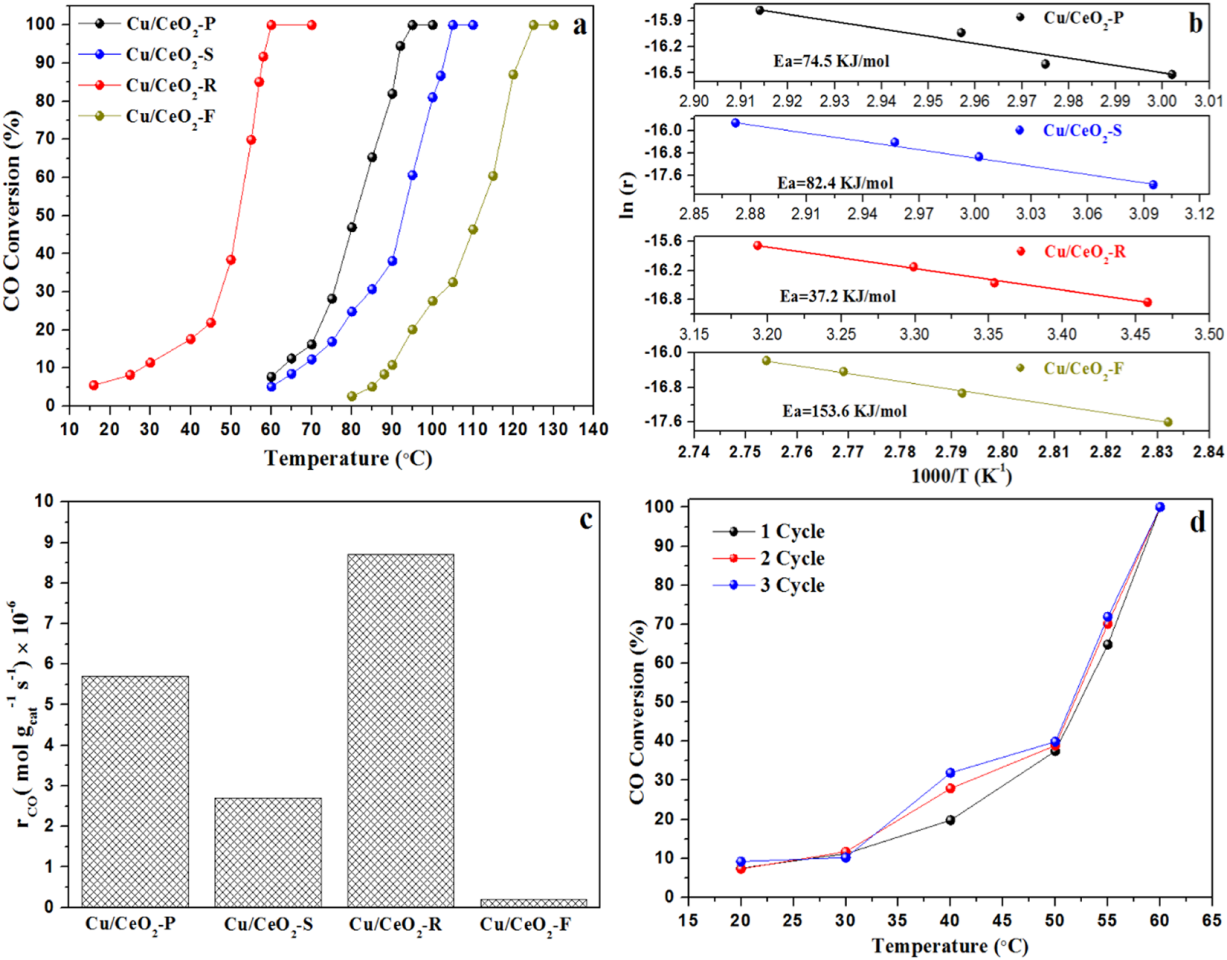
CO conversion (**a**), Arrhenius plots of CO reaction under CO conversion below 10% (**b**), CO reaction rate (r_CO_) derived from Arrhenius plots (**c**) of these Cu/CeO_2_ catalysts with different morphology, the cyclic stability of Cu/CeO_2_-R catalyst (**d**).

As we all know, the morphology of catalysts would greatly affect its catalytic performance, thus we take CO oxidation as an example to evaluate the catalytic activity and stability of these Cu/CeO_2_ catalysts. According to Fig. 1a, the Cu/CeO_2_-R catalyst exhibited better activity than other three catalysts, with 90% CO conversion at 58 °C, which was substantially lower than the corresponding temperature for the Cu/CeO_2_-F catalyst (T_90_ = 121 °C). In addition, it was also discovered that the complete conversion temperature of CO over Cu/CeO_2_-P catalyst at 91 °C was close to that of Cu/CeO_2_-S catalyst at 103 °C.

Figure 1b showed Arrhenius plots of CO conversion with respect to the inverse reaction temperature. The apparent reaction barriers (Supporting Information) are derived as 37.2 kJ mol^−1^, 74.5 kJ mol^−1^, 82.4 kJ mol^−1^ and 153.6 kJ·mol^−1^ for the Cu/CeO_2_-R, Cu/CeO_2_-P, Cu/CeO_2_-S and Cu/CeO_2_-F catalysts, respectively. All the reaction results indicated that the CeO_2_ nanorods covered with Cu NPs exhibited the best CO oxidation activity. The introduction of CeO_2_ nanorods greatly decreased the energy barrier of CO oxidation reaction, while the using of flower-like CeO_2_ microspheres resulted in the higher energy barrier of CO oxidation.

Besides, in Fig. 1c the CO oxidation reaction rate for Cu/CeO_2_-R, Cu/CeO_2_-P, Cu/CeO_2_-S and Cu/CeO_2_-F are respectively 8.7 × 10^−6^ mol g_cat_^−1^ s^−1^, 5.6 × 10^−6^ mol g_cat_^−1^ s^−1^, 2.7 × 10^−6^ mol g_cat_^−1^ s^−1^, and 0.2 × 10^−6^ mol g_cat_^−1^ s^−1^. The CO reaction rate of Cu/CeO_2_-R catalyst is about 40 times higher than that of Cu/CeO_2_-F catalyst, indicating that the morphology of Cu/CeO_2_ catalysts greatly affected their catalytic performance.

The thermal stability is very important for the practical application of catalyst, and thus the cycle stability of Cu/CeO_2_-R catalyst is tested at different temperature. According to Fig. 1d, the Cu/CeO_2_-R catalyst was tested through three runs, and it was discovered that CO conversion at the third run still kept almost equivalent with the first run. These results verified that the Cu/CeO_2_-R catalyst owned the superior thermal stability.

**Figure 2:**
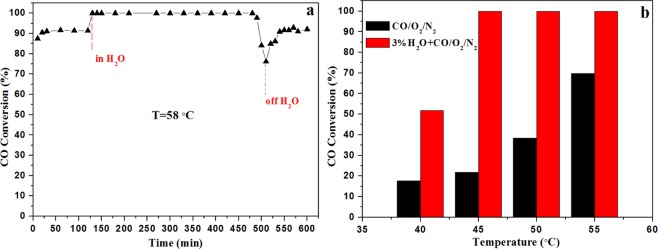
Water resistance (**a**), the promotion role of H_2_O (**b**) over the Cu/CeO_2_-R catalyst.

For practical circumstance, there is generally a small amount of H_2_O vapor in the feed gas. Thus, the H_2_O resistance was performed at 58 °C on the best Cu/CeO_2_-R catalyst (Fig. 2a). Interestingly, it could be seen that the introduction of 3% H_2_O at 58 °C resulted in an obvious increase of CO conversion from 90% to 100% over Cu/CeO_2_-R catalyst. Simultaneously, a 100% conversion of CO could keep 9 h and then exhibited a decline. When the CO conversion decreased to 75% in the present of H_2_O, we turned off H_2_O and it resulted in a gradual increase of CO conversion, indicating that H_2_O played the significant role in promoting the conversion of CO. However, the excessive present of H_2_O would lead to the deactivation of Cu/CeO_2_-R catalyst, and the reasons might be related to the comparative adsorption of H_2_O and CO on the surface of catalysts^41^. A. Martínez-Arias *et al*.^42^ reported that the deactivation of a CuO/CeO_2_ catalyst under humidity conditions CO oxidation are mainly related to the modifications of interfacial sites due to the formation of specific carbonates and a blocking effect induced by the presence of adsorbed molecular water, respectively. The decrease of active sites could limit the catalytic activity of CO oxidation. Accordingly, the dramatic fast drop of CO conversion after 9 hours in this work might be related to a blocking effect induced by the presence of excessively adsorbed water molecules. It was also easy to understand about a gradual increase of CO conversion after stopping water, and the excessively adsorbed molecular water was slowly consumed, thereby weakening the competitive adsorption capacity of the active sites to expose more active sites for adsorption and activation of CO and O_2_ molecules.

To define the specific promotion degree of H_2_O, in further we investigated the effect of H_2_O at the different reaction temperature and compared CO conversion with respect to temperature. According to Fig. 2b, we discovered that the CO conversion increased from 18% to 54% at 40 °C under humidity, while the further increase of reaction temperature led to the obvious improvement of CO conversion. For example, the reaction temperature increased from 40 °C to 45 °C, which resulted in the extremely significant increase of CO conversion from 24% to 100%. For Fig. 2b, it was discovered that the promotion of H_2_O became more and more critical with the increase of reaction temperature.

### Texture properties and Morphology of these Cu/CeO_2_ catalysts

The textural properties of CeO_2_ and Cu/CeO_2_ samples were explored from their corresponding N_2_ adsorption-desorption isotherms at −196 °C. The BET specific surface area and cumulative pore volume were summarized in Tables 1 and S1. As shown in Fig. S1a, the isotherm of all CeO_2_ and Cu/CeO_2_ samples exhibited the reversible type IV isotherm, according to the IUPAC classification. The characteristic of all CeO_2_ and Cu/CeO_2_ catalysts with an important contribution of mesoporous was exhibited in Fig. S1b, which was concluded from the sharp increase of the pore volume at low p/p_0_. Besides, BET specific surface area of these Cu/CeO_2_ catalysts is ranging between 88.6 m^2^ g^−1^ and 28.5 m^2^ g^−1^, and the reason is most probably attributable to the size difference of CeO_2_ and a certain degree of pore blockage caused by the presence of copper species on the surface of CeO_2_.

**Table 1:** Texture characteristics of the Cu/CeO_2_ catalysts with different morphology.

Catalysts	S_BET_^*a*^ (m^2^/g)	D_p_^*a*^ (nm)	V_p_^*a*^ (cm^3^/g)	d_CeO2_^*b*^ (nm)	d_CuO_^*b*^ (nm)	d_CeO2_^*c*^ (nm)	d_CuO_^*c*^ (nm)	Cu loading^*d*^ (wt %)	H_2_ uptake^*e*^ (µmol H_2_/g_cat_)
Cu/CeO_2_-P	76.4	7.29	0.18	9.84	—	19	7.5	12.96	187
Cu/CeO_2_-S	88.6	8.60	0.23	9.48	—	22	11.0	15.07	154
Cu/CeO_2_-R	84.7	10.88	0.29	9.65	—	15	6.0	12.34	167
Cu/CeO_2_-F	28.5	10.44	0.11	17.14	19.46	40	23.0	12.62	131

^*a*^The BET surface area, pore volume, and pore size were determined by N_2_ physical-adsorption, ^*b*^Crystal size of CeO_2_ (111) and CuO was calculated according to Scherrer equation, ^c^Particles size of CeO_2_ and CuO was calculated according to TEM analysis (Fig. S9), ^*d*^Element content was measured by ICP-OES, ^*e*^Actual amount of hydrogen consumption was obtained by H_2_-TPR.

**Figure 3:**
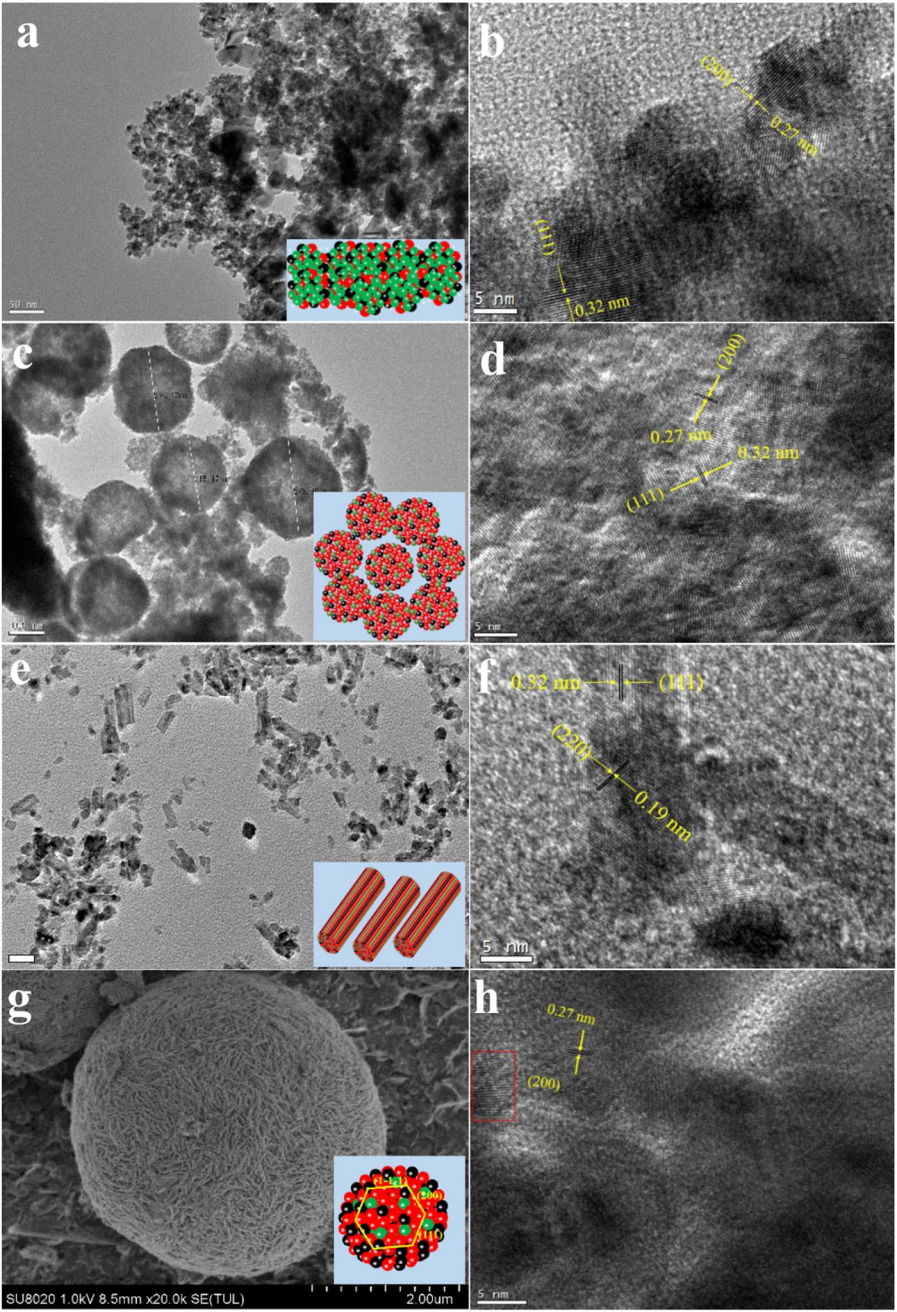
SEM, TEM and HRTEM patterns of the Cu/CeO_2_ catalysts: (**a**) TEM of Cu/CeO_2_-P catalyst, (**b**) HRTEM of Cu/CeO_2_-P catalyst; (**c**) TEM of Cu/CeO_2_-S catalyst, (**d**) HRTEM of Cu/CeO_2_-S catalyst; (**e**) TEM of Cu/CeO_2_-R catalyst, (**f**) HRTEM of Cu/CeO_2_-R catalyst; (**g**) SEM of Cu/CeO_2_-F catalyst, (**h**) HRTEM of Cu/CeO_2_-F catalyst.

The morphology of CeO_2_ not only affected the exposed crystal plane, but also modulated the size of supported CuO NPs as it was treated as support. Herein, to explore the relation of morphology and the exposed crystal plane, SEM, TEM and HRTEM analysis were conducted. According to Fig. 3, we discovered that these Cu/CeO_2_ catalysts formed their unique morphology, such as a Cu/CeO_2_-R with nanorods shape, a Cu/CeO_2_-S with nanospheres shape, a Cu/CeO_2_-P with nanoparticles shape and a Cu/CeO_2_-F with flower-like microspheres shape. The Cu/CeO_2_-P catalyst was constructed by a large number of small nanoparticles with average sizes of around 10–20 nm (Figs 3a and S2), and the exposed crystal planes were related to (200) and (111) with inter-planar spacings of 0.27 and 0.32 nm, respectively (Figs 3b and S3). The Cu/CeO_2_-S catalyst was composed of nanospheres with diameter of 250 nm (Figs 3c and S4), and the HRTEM images verified that the Cu/CeO_2_-S also exposed the (200) and (111) crystal planes with interplanar spacings of 0.27 and 0.32 in Figs 3d and S5. The Cu/CeO_2_-R catalyst derived from lots of nanorods with lengths of 30–100 nm (Figs 3e and S6), and the HRTEM images displayed the (220) and (111) lattice fringes with the inter-planar spacing of 0.28 and 0.19 nm (Figs 3f and S6). For Figs 3g and S8, the Cu/CeO_2_-F catalyst was composed of flower-like microspheres with the diameter of 4 µm, and its HRTEM images exhibited the (200) crystal planes with inter-planar spacings of 0.27 nm (Figs 3h and S9). Therefore, the above results suggested these Cu/CeO_2_ catalysts with different morphology exposed the different crystal planes: the {111} and {100} crystal planes for Cu/CeO_2_-P and Cu/CeO_2_-S catalysts, the {110} crystal planes for Cu/CeO_2_-R catalyst, and the {100} crystal planes for Cu/CeO_2_-F catalyst. Combined with their activity, it was considered that the exposed (110) crystal planes of CeO_2_ nanorods might be beneficial to promote the conversion of CO.

### Size effect of CeO_2_ and Cu nanoparticles (NPs)

**Figure 4:**
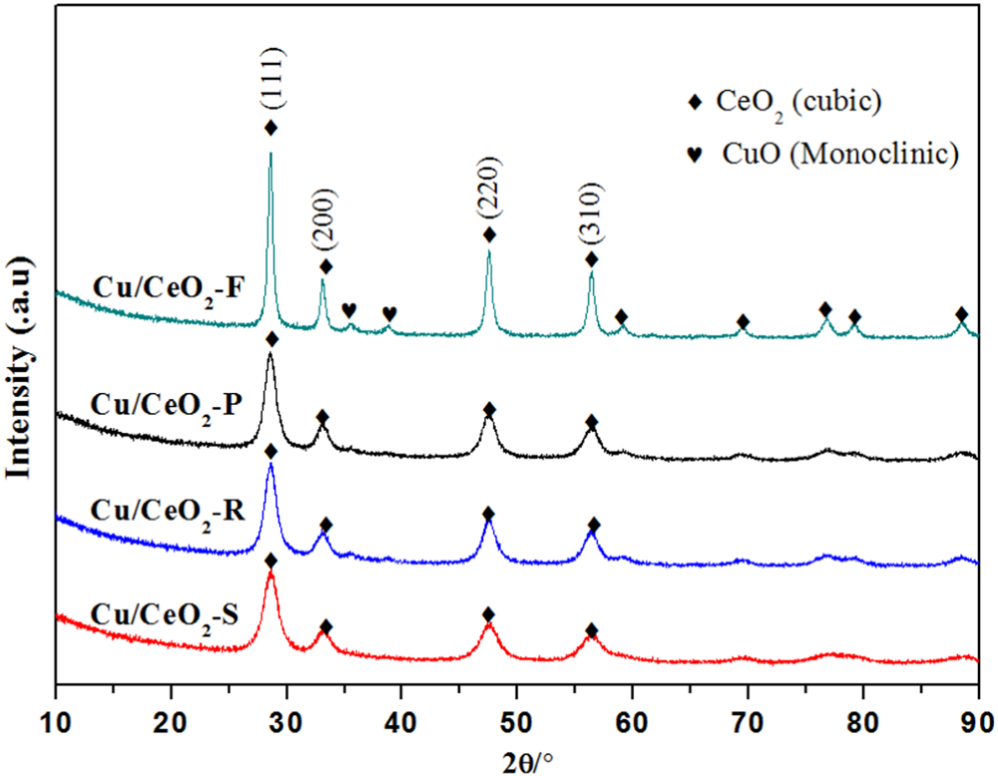
XRD patterns of the Cu/CeO_2_ catalysts with different morphology.

Figure 4 shows the XRD patterns of Cu/CeO_2_ catalysts with different morphology. According to Fig. 4, these Cu/CeO_2_ samples exhibited the diffraction peaks at 28.5°, 33.1°, 47.5° and 56.3°, indicating that the main diffraction peaks of these Cu/CeO_2_ samples matched with the fluorite-type cubic CeO_2_ phase (JCPDS 89–8436). The crystal size was calculated according to Scherrer equation, and half width of CeO_2_ (111) plane at 2θ = 28.5° was selected. The half width at 28.5° follows the order of Cu/CeO_2_-S < Cu/CeO_2_-R < Cu/CeO_2_-P < Cu/CeO_2_-F (Table 1), indicating that the morphology and texture properties of CeO_2_ support greatly altered its crystal size. Notably, in Fig. 4 the Cu/CeO_2_-F sample displayed two weak diffraction peaks of CuO (JCPDS 80–1268) at 2θ of 35.6° and 38.7°, and the Cu/CeO_2_-R sample also displayed two extremely weak diffraction peaks of CuO at 35.6° and 38.7°, indicating that copper oxide crystal size of Cu/CeO_2_-R was further smaller than that of Cu/CeO_2_-F. However, it is no obvious CuO diffraction peak for Cu/CeO_2_-S and Cu/CeO_2_-P, which was possibly related to the contribution of the ultra-small CuO grain on these two catalysts. Therefore, it was concluded that the difference of CeO_2_ morphology and crystal size might also lead to the growth difference of CuO grain (Fig. S10). Combined with the reaction rate of CO (r_co_) on these Cu/CeO_2_ catalysts, it was discovered that the lower r_co_ of Cu/CeO_2_-F catalyst might be due to the larger CeO_2_ and CuO crystal size, which decreased the dispersion of CuO NPs and inhibited the conversion of CO.

**Figure 5:**
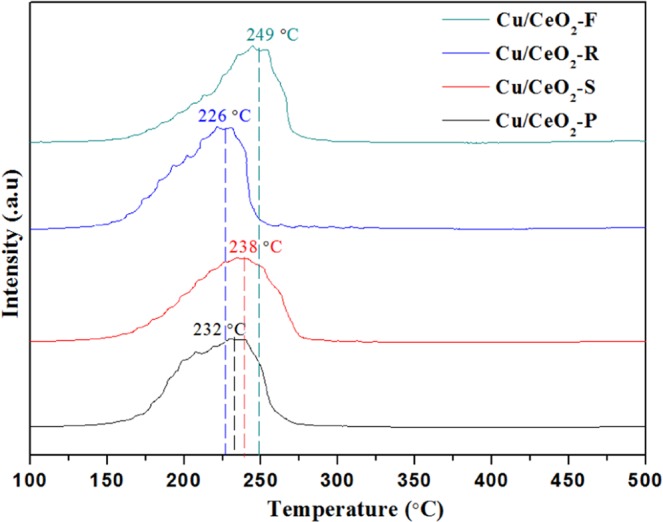
H_2_-TPR patterns of the Cu/CeO_2_ catalysts with different morphology.

In order to verify the reducibility of these Cu/CeO_2_ catalysts, H_2_-TPR analysis was conducted, and the results shown in Fig. 5. It was discovered that Cu/CeO_2_-R catalyst exhibited an asymmetric reduction peak at 226 °C, and Cu/CeO_2_-P catalyst also showed an asymmetric reduction peak at 232 °C. Besides, the Cu/CeO_2_-S and Cu/CeO_2_-F catalysts respectively appeared asymmetric reduction peak at 236 °C and 249 °C. These asymmetric reduction peaks might be related to the different particles size of CuO NPs and the interaction of Cu species and CeO_2_ support. The former works^43,44^ reported that the small CuO particles are more easily reduced than large CuO particles. Therefore, it was verified that the Cu/CeO_2_-F catalyst owned the larger CuO particles. Similarly, the particles size of CuO on these catalysts is in the order of Cu/CeO_2_-F > Cu/CeO_2_-S > Cu/CeO_2_-P > Cu/CeO_2_-R catalysts on basis of H_2_-TPR analysis. The smaller CuO NPs can accelerate the oxidation of CO^45^, which may be one of the reasons that Cu/CeO_2_-R catalyst exhibited the best activity in CO oxidation reaction. Besides, the strong interaction of Cu species and CeO_2_ support (Ce^4+^ + Cu^+^ → Ce^3+^ + Cu^2+^) was advantageous to promoting the dispersion of copper species, and then it might be considered that the Cu/CeO_2_-R catalyst had a stronger interaction between Cu species and CeO_2_ nanorods. The H_2_-TPR results are consistent with CO-TPR (Fig. S11). Besides, the reduction peak above 300 °C was not experimentally included, and then we assumed that the surface oxygen originated from the complete reduction of Cu^2+^ to Cu°. In order to quantitatively determine the quality hydrogen consumption, a series of pure CuO samples are treated as the reference, and the H_2_ uptake (µmol H_2_/g_cat_) of these Cu/CeO_2_ catalysts is calculated by the external standard method in Table 1. On basis of standard curve for hydrogen consumption of different masses of CuO, the amount of H_2_ assumption over these Cu/CeO_2_ samples follows the order of Cu/CeO_2_-P > Cu/CeO_2_-R > Cu/CeO_2_-S > Cu/CeO_2_-F, which is attributed to the different amounts of oxygen derived from the bulk and surface Cu_x_O (x ≤ 2).

### The surface chemical properties and lattice defects

**Figure 6:**
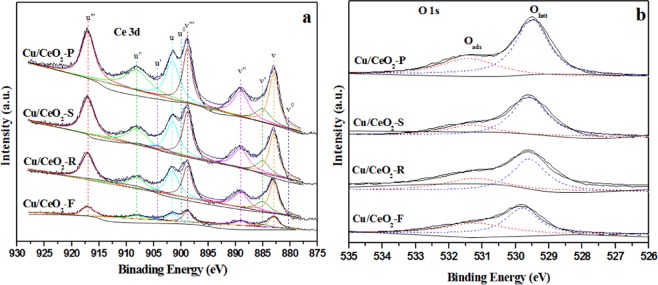
(**a**) Ce 3d and (**b**) O1s XPS analysis of these Cu/CeO_2_ catalysts.

In order to obtain the chemical state and the surface composition information of these Cu/CeO_2_ catalysts, the XPS characterization was performed. Figure 6a showed the Ce 3d XPS spectra of Cu/CeO_2_ catalysts. Accordingly, it was divided into the ten peaks corresponding to five pairs of Ce 3d spin-orbit doublets. There are two types spin-orbit components, including the Ce 3d_3/2_ (marked as u_0_-u”’) and the Ce 3d_5/2_ (marked as v_0_-v”’). Generally, oxygen vacancies were formed in the CeO_2_ fluorite lattice to maintain charge balance, and the number of oxygen vacancies affected the adsorption properties of Ce-based catalysts. For Fig. 6a, the peaks of u°, u’, v° and v’ were related to the 3d_3/2_ and 3d_5/2_ of Ce^3+^ species, and other peaks were ascribed to the Ce^4+^ species^46–49^. The relative content of Ce^3+^ species can be calculated on basis of peak area ratio of u°, u’, v° and v’ to the total peak area of Ce 3d (Table 2). The production of surface oxygen vacancy derived from the removal of lattice oxygen (O_latt_) species in CeO_2_ and resulted in the formation of Ce^3+^ species (Ce^4+^ + e^−^ → Ce^3+^ + ◇), since the content of Ce^3+^ species can semi-quantitatively determine the surface oxygen vacancy. According to Table 2, the surface content of Ce^3+^ species is in the order of Cu/CeO_2_-F > Cu/CeO_2_-R > Cu/CeO_2_-S > Cu/CeO_2_-P catalysts, indicating that the Cu/CeO_2_-F and Cu/CeO_2_-R catalysts owned the relative more surface oxygen vacancies. Combined with Figs 1a and 6a, it was discovered that the CO oxidation activity might be also related to the amount of surface oxygen vacancies (Ce^3+^ species), indicating that surface oxygen vacancies played the key role in determining the conversion of CO. However, due to the lower dispersion of CuO species over the Cu/CeO_2_-F catalyst exhibited the lower CO oxidation activity, indicating the Ce^3+^ species and the dispersion of CuO species both affected the activity of Cu/CeO_2_ catalyst.

**Table 2:** Chemical properties of the Cu/CeO_2_ catalysts with different morphology.

Catalysts	the exposed crystal planes of CeO_2_^*a*^	Ce^3+^/{Ce^3+^ + Ce^4+^}^*b*^	O_latt_/{O_latt_ + O_ads_}^*b*^	Cu/Ce^c^	I_D_/I_F2g_^*d*^	T_90_^*e*^(°C)
Cu/CeO2-P	(100) (111)	5.97	49.48	1.34	0.65	91
Cu/CeO2-S	(100) (111)	8.52	49.43	1.02	0.36	103
Cu/CeO2-R	(110) (111)	8.67	49.52	1.81	0.68	58
Cu/CeO2-F	(100)	9.11	48.57	3.37	0.13	121

^*a*^The exposed crystal planes of CeO_2_ according to HRTEM analysis, ^*b*^amount of per elements on the surface of samples was obtained by XPS characterization, ^*c*^the atomic ratio of Cu/Ce was obtained by XPS analysis, ^*d*^the amount of defect sites was evaluated through Raman characterization, ^*e*^reaction temperature reaction tem perature at 90% CO conversion.

The XPS spectra in the O 1 s region can also be deconvoluted into two peaks in Fig. 6b. According to former reports^50,51^, the peak with high binding energy was related to the surface adsorbed oxygen species (denoted as O_ads_), and the peak with low binding energy was assigned to surface lattice oxygen species (denoted as O_latt_). The binding energy of the lattice oxygen (O_latt_) in Ce (IV) oxide was about 529.6 eV. The binding energy of O_ads_ on the surface of ceria oxide, such as hydroxyl groups on the surface, oxygen chemisorbed on the surface, grain-boundary impurities and oxide ions in the defective CeO_x_ (x < 2) was about 531–533 eV^52–54^. Yao. K *et al*.^55–57^ also reported that the binding energy of lattice oxygen (O_latt_) in Cu_2_O phase shifted to 530.3 eV, the binding energy of the lattice oxygen (O_latt_) in CuO phase was related to 529.3–529.6 eV, and the binding energy of the oxygen (O_ads_) adsorbed on the surface of CuO and -Cu_2_O phase was ascribed to at 531.5 eV. Accordingly, it was concluded that in this work the binging energy of peaks was close to that of lattice oxygen in CuO and Ce (IV) oxide, indicating that CuO and Ce (IV) oxide phases were formed in these Cu/CeO_2_ catalysts. Generally, for CO oxidation reaction, the O_latt_ species were considered as the active oxygen^58^. Herein, we calculated the content of O_latt_ species according to the peak area, and the results in Table 2 showed that the content of O_latt_ follows in order of Cu/CeO_2_-R > Cu/CeO_2_-P > Cu/CeO_2_-S > Cu/CeO_2_-F. Generally, the removal of O_latt_ species in CeO_2_ would form a large number of oxygen vacancies, which is advantageous to promoting the conversion of CO.

**Figure 7:**
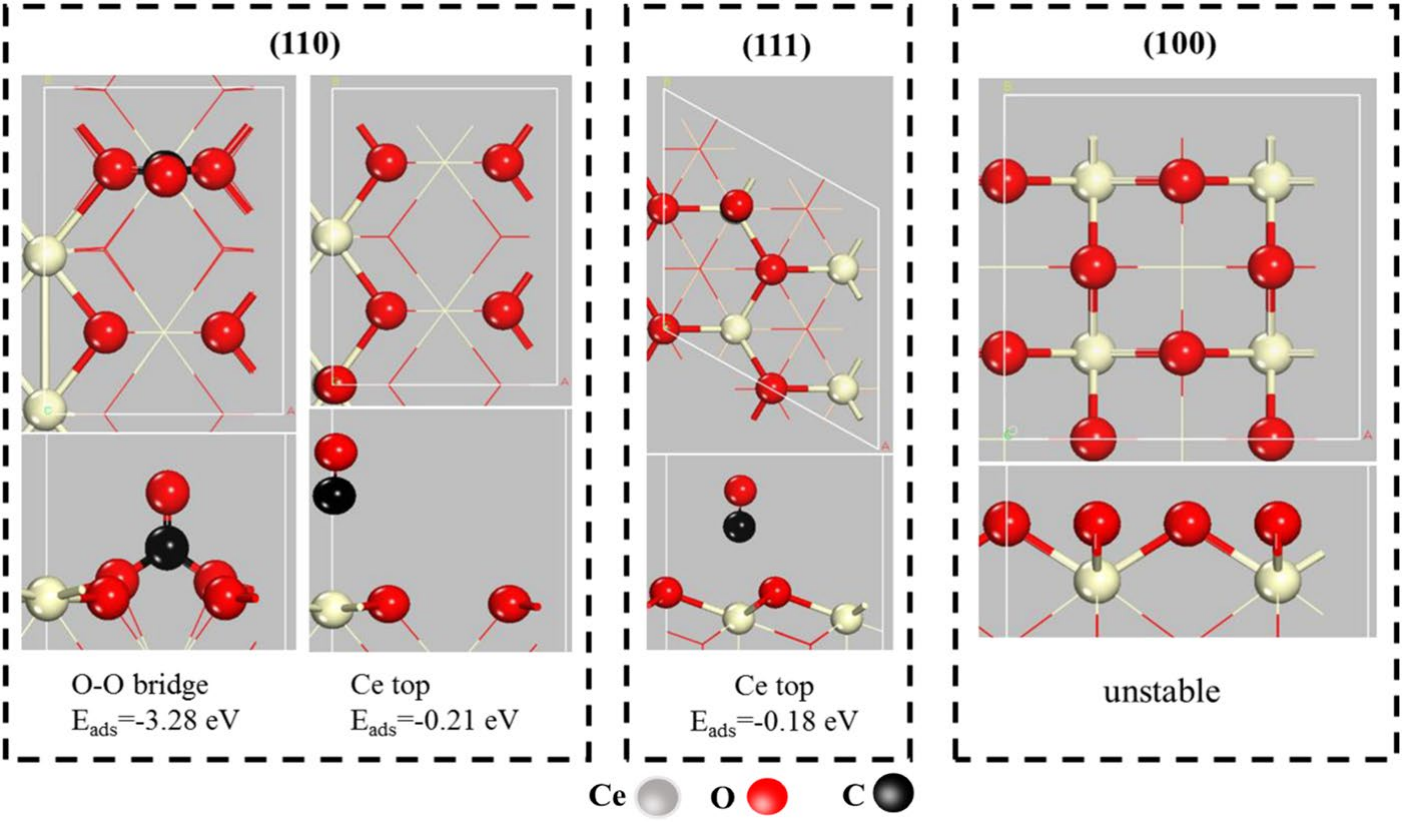
Adsorption of CO molecule at the different crystal planes of pure CeO_2_.

In order to theoretically explore the role of oxygen vacancy on CeO_2_ surface, a density functional theory (DFT) calculation was also conducted. The calculation was done by density functional theory (DFT) method employing the VASP package with PBE + U (Ueff = 5.0 eV) approximation^59–61^. First, CO adsorption energy on the CeO_2_ (110), (111) and (100) surface without oxygen vacancy was systematically investigated, and the results shown in Fig. 7. The adsorption of CO on the clean CeO_2_ (110) surface existed two models, including the adsorption on the O-O bridge site and the top of Ce. The adsorption energy on these two models of the clean CeO_2_ (110) surface was calculated, and the E_ads_° was respectively −3.28 eV and −0.21 eV in Table 3. Besides, the adsorption energy of CO on the clean CeO_2_ (111) surface is −0.18 eV, which was considered on the top of Ce atom. Similarly, it was also found that CO adsorption on the clean CeO_2_ (100) surface was greatly unstable. When the oxygen termination is selected, CO can immediately react with the oxygen to form CO_2_.

**Table 3:** Adsorption energy of CO molecular and Formation energy of oxygen vacancies on the top of CeO_2_.

Crystal planes	E_ads_^0*a*^	E_(F)_^*b*^	E_ads_^*c*^
(111)	−0.18	−0.26	−0.67
(110)	−3.28/−0.21	−1.35	−3.78/−0.21
(100)	Unstable	−10.62	−0.33/−0.04

aCO adsorption energy on the clean CeO_2_ surface without oxygen vacancies, ^b^the formation energy of oxygen vacancies at the CeO_2_ surface, ^c^CO adsorption energy on the CeO_2_ surface with oxygen vacancies.

**Figure 8:**
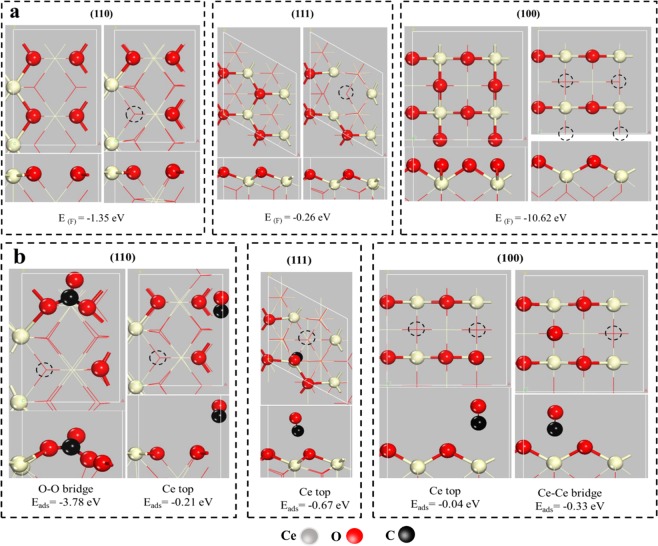
(**a**) Formation energy of oxygen vacancies on the different crystal planes of CeO_2_, (**b**) Adsorption of CO molecule at the different crystal planes of CeO_2_ surface with oxygen vacancies.

Comparatively, the adsorption energy of CO on the CeO_2_ (110), (100) and (111) surface with oxygen vacancy is also investigated using DFT method. Before this, the formation energy on the CeO_2_ (110), (111) and (100) surface with oxygen vacancy is necessarily explored, and the results shown in Fig. 8a and Table 3. The adsorption of CO on the CeO_2_ (110) surface containing oxygen vacancy also existed two models, including the adsorption on the O-O bridge site and the top of Ce atom. The adsorption energy of CO at the O-O bridge site of CeO_2_ (110) surface is −3.78 eV, which is higher than that of the clean CeO_2_ (110) surface. The adsorption energy of CO at the top of Ce atom is −0.21 eV, which is the same to that of the clean CeO_2_ (110) surface. In addition, the adsorption energy of CO on the CeO_2_ (111) surface is −0.67 eV, which is lower than that of the clean CeO_2_ (111) surface. Besides, it is found that CO adsorption energy on the CeO_2_ (100) surface is −0.33 eV, indicating that the existence of oxygen vacancy decrease CO adsorption energy of CeO_2_ (100) surface. Interestingly, the formation of oxygen vacancy on the CeO_2_ (110) surface could enhance the adsorption of CO due to the decrease of adsorption energy, which is vitally significant for promoting the conversion of CO. Combined with Figs 1, 3 and 8, it is verified that the CeO_2_ (110) crystal plane is advantageous to promote CO oxidation, which is also one of the reasons that Cu/CeO_2_-R sample exhibited the superior performance in CO oxidation reaction.

**Figure 9:**
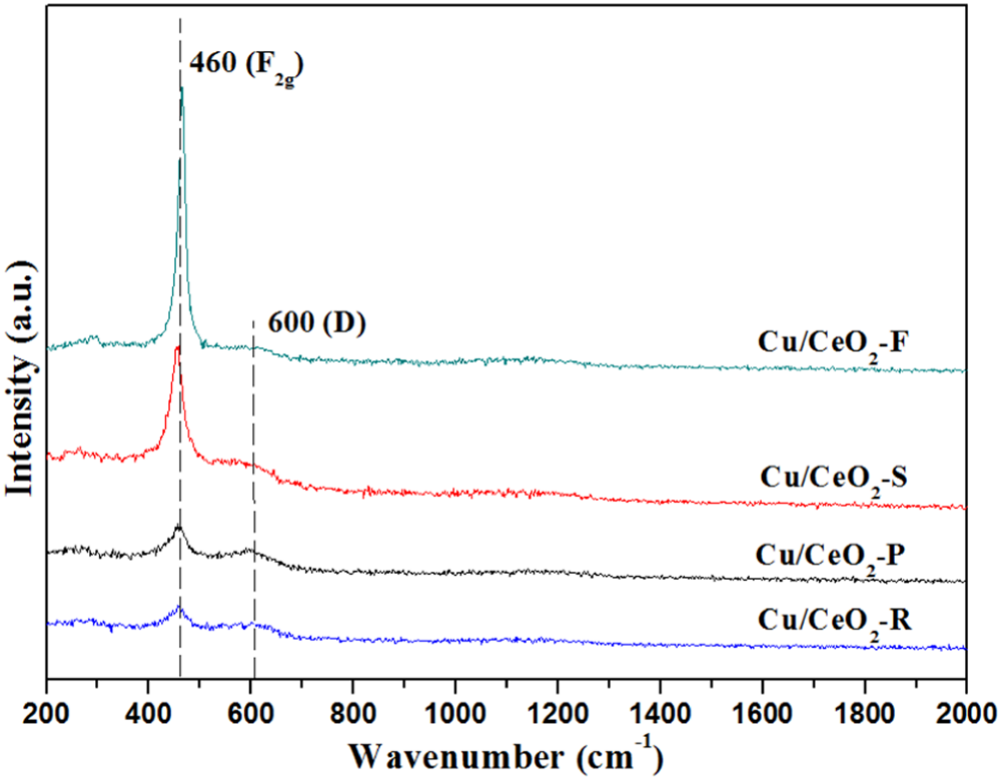
Raman spectra of the Cu/CeO_2_ catalysts with different morphology.

Raman characterization was performed on these CeO_2_ and Cu/CeO_2_ catalysts to further investigate their surface information. As shown in Figs 9 and S13, the peak centered at 458 cm^−1^ in Raman spectra was ascribed to the F_2g_ symmetric vibration (Ce-O-Ce stretching). According to work^62^, the peak centered at near 464 cm^−1^ was related to the pure cubic fluorite CeO_2_. The obvious red shift of F_2g_ peak in these Cu/CeO_2_ samples indicated the change of surface crystal lattice parameter in CeO_2_^63^. Besides, a broad peak of the Raman spectra centered at 600 cm^−1^ is due to the existence of oxygen vacancy on the surface of these Cu/CeO_2_ catalysts^64^. The relative amount of oxygen vacancy can be calculated as intensity ratio of bands centered at 600 cm^−1^ and 458 cm^−1^ (I_D_/I_F2g_)^27^, also indicating the relative amount of surface lattice defect site, and the results were summarized in Table 2. It was discovered that the I_D_/I_F2g_ ratio of the Cu/CeO_2_-R catalyst was higher, indicating the relative amount of surface lattice defect site was more. Besides, the I_D_/I_F2g_ ratio of Cu/CeO_2_-P catalyst is very close to that of Cu/CeO_2_-R catalyst. The I_D_/I_F2g_ ratio indicated that the morphology of Cu/CeO_2_ catalysts can greatly modulate the surface lattice defect sites due to the strong interface interaction between CuO NPs and CeO_2_. The above results suggested that the CeO_2_ nanorods as support can notably promote the production of more surface lattice defect sites.

**Figure 10:**
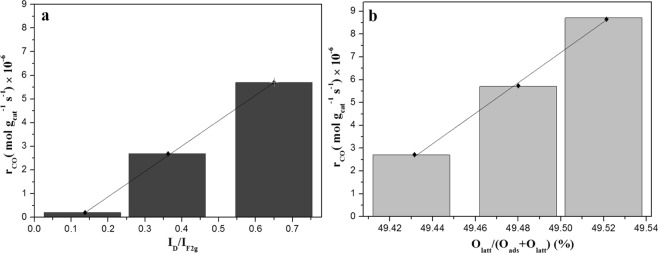
The relation of (**a**) surface lattice defect sites and the reaction rate of CO, (**b**) surface lattice oxygen species and the reaction rate of CO (r_CO_).

The relation of surface lattice defect sites and CO activity on these Cu/CeO_2_ catalysts was associated in Fig. 10. Through comparing the relation of surface lattice defect sites derived from Raman analysis and r_CO_ in Fig. 10a, we discovered that the surface lattice defect sites was also a factor in promoting the conversion of CO, and the amount of surface lattice defect sites was positively related to r_CO_. Besides, in Fig. 10b, the relation of lattice oxygen species (O_latt_) derived from O1s XPS was also positively related to the activity of Cu/CeO_2_ catalyst in CO oxidation reaction. Therefore, the increase of surface lattice defect sites would be beneficial to improving the catalytic performance of Cu/CeO_2_ catalysts, which can modulate the adsorption properties of reactant molecular.

**Figure 11:**
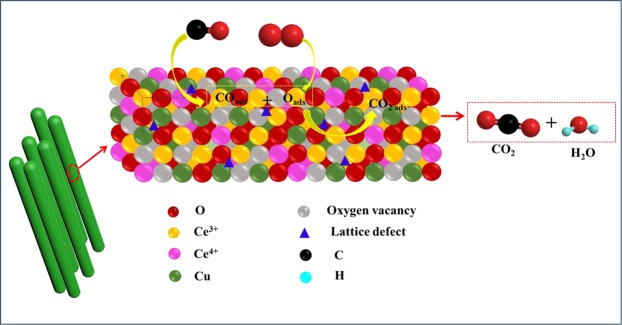
A proposed reaction pathway for CO oxidation over Cu/CeO_2_ catalysts.

Combined with a series of characterizations and DFT calculation, it was verified that morphology of CeO_2_ played the key role in determining the dispersion of supported Cu NPs, its exposed crystal plane, the interaction of Cu species and CeO_2_ support, the number of O_latt_ species and oxygen vacancies, which were vitally important for improving the catalytic performance of Cu/CeO_2_ catalysts. For example, former works^38,65,66^ considered that the Ce-based catalysts with exposed CeO_2_ (111)/(100) planes owned much higher activity in comparison to the Ce-based catalysts exposed by CeO_2_ (110)/(100) planes, indicating that the exposed plane was vitally important for determining the catalytic activity of Ce-based catalyst. Simultaneously, we discovered that the lattice oxygen species (O_latt_) exhibited the effect on catalytic performance of Cu/CeO_2_ catalysts in CO oxidation reaction, and then a reaction pathway was proposed over Cu/CeO_2_ catalysts in Fig. 11. Combined with the O 1s XPS characterization, it was verified the Cu/CeO_2_-R catalyst exhibited the more O_latt_ species, which might be the main reason with the better activity in CO oxidation reaction. The oxidation of CO over the Cu/CeO_2_-R catalyst is as follows: CO molecular first adsorbed the metal active sites, and thus reacted with the adjacent lattice oxygen (O_latt_) to form CO_2_, H_2_O and oxygen vacancy. Afterwards, O_2_ molecular was absorbed and replenished into this oxygen vacancy, and reacted with another CO molecular. So far, a redox cycle was completed. A large number of works^67,68^ also reported that oxygen vacancies are very significant for promoting the conversion of CO. Therefore, the morphology and size of CeO_2_ could not only modulate the dispersion of supported metal, but also alter the interaction of Cu species and CeO_2_ support (Ce^4+^ + Cu^+^ → Ce^3+^ + Cu^2+^), the amount of lattice oxygen (O_latt_) species and lattice defect sites, which played the important role in determining the reaction rate of CO.

### The promoting role of H_2_O on the catalytic performance of Cu/CeO_2_-R catalyst

**Figure 12:**
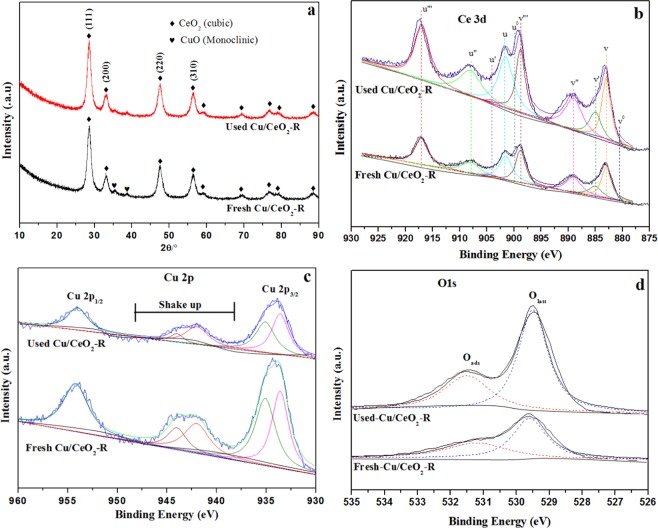
The structure and chemical properties of the Cu/CeO_2_-R catalyst before and after H_2_O resistance reaction: (**a**) XRD analysis; (**b**) Ce 3d XPS; (**c**) Cu 2p XPS; (**d**) O 1sXPS.

It has been verified that Cu/CeO_2_-R catalyst owned the best catalytic performance in CO oxidation reaction, and the excellent H_2_O resistance was also discovered. To explore the promoting role of H_2_O, the used Cu/CeO_2_-R catalyst was systematically analyzed on basis of a series of characterizations, and the results were listed in Fig. 12. It was discovered that the XRD of used Cu/CeO_2_-R catalyst is the same to that of fresh Cu/CeO-R catalyst, indicating that its phase composition remained unchanged before and after H_2_O resistance reaction. Interestingly, the XPS results verified that the Ce^3+^ and O_latt_ species of used Cu/CeO_2_-R catalyst are obviously higher than that of fresh Cu/CeO_2_-R catalyst, suggesting that the adsorption of H_2_O in the surface of Cu/CeO_2_-R catalyst can promote the formation of surface Ce^3+^ species. More importantly, the results of Raman also verified that the obvious increase of surface lattice defect sites was discovered on used Cu/CeO_2_-R catalyst in Fig. S14. Besides, the H_2_-TPR characterization of Cu/CeO_2_-R catalyst after H_2_O resistance reaction was also investigated (Fig. S15), and the results is consistent with XRD results. The above results confirmed that the formation of Ce^3+^ species and the increase of surface lattice defect sites in humidity conditions were the main factors to strengthen the H_2_O resistance of Cu/CeO_2_-R catalyst.

## Conclusion

In summary, Cu/CeO_2_ catalysts with different morphology and size have been successfully synthesized by hydrothermal and solvothermal methods, and followed by deposition precipitation process. Notably, these catalysts were studied for the catalytic oxidation of CO under dry and humid conditions to explore the shape effect on CO oxidation performance. The results verified that the complete conversion temperature of CO was 60 °C on Cu/CeO_2_-R catalyst, 90 °C on Cu/CeO_2_-P catalyst, 105 °C on Cu/CeO_2_-S catalyst and 125 °C on Cu/CeO_2_-F catalyst, respectively. Based on a series of characterizations, it was concluded that the Cu/CeO_2_-R catalyst exposed the highly active CeO_2_ (110) crystal plane, owned the smaller particle size of CeO_2_ and CuO, formed the stronger interaction between Cu species and CeO_2_ nanorods (Ce^4+^ + Cu^+^ → Ce^3+^ + Cu^2+^), and formed a large number of oxygen vacancies derived from the removal of lattice oxygen (O_latt_) species in CeO_2_ and the lattice defect sites, which jointly promoted the conversion of CO at low temperature (T_90_ = 58 °C). In addition, the presence of humidity greatly improved the activity of Cu/CeO_2_-R catalyst, exhibited an obvious increase of CO conversion at the same conditions and required for the reaction to reach 100% CO conversion at the lower temperature. Therefore, Cu/CeO_2_-R catalyst is a promising candidate for the elimination of CO in exhaust gas streams. Simultaneously, this work also give further insight for understanding the catalytic role of the exposed crystal plane, the crystal size, surface oxygen vacancies and lattice defects, and provide some guidance for the rational design and synthesis of catalysts with the metal/oxide configuration.

## Experimental Sections

### General considerations

All the solvents and reagents were of analytical grade and were used as source. Cetyltrimethyl ammonium bromide (CTAB), glucose, acrylamide, Ce(NO_3_)_3_·6H_2_O, Cu(NO_3_)_2_·3H_2_O, NaOH and anhydrous sodium carbonate were purchased from Shanghai Aladdin Reagent Co Ltd. In general, all of the chemicals were used as source without further purification.

### Preparation of the Cu/CeO_2_ catalyst with the different morphology

At first, the CeO_2_ support with the different morphology and size was synthesized, and the detailed preparation process of CeO_2_ nanoparicles **(CeO**_**2**_**-P)**, CeO_2_ nanosphere **(CeO**_**2**_**-S)**, CeO_2_ nanorods (**CeO**_**2**_**-R**) and flowerlike CeO_2_ microspheres (**CeO**_**2**_**-F**) was listed in Supporting Information.

For the synthesis of the Cu/CeO_2_ catalysts, the CeO_2_ powders (1.0 g) were suspended in 50 mL of deionized water under vigorous stirring. 1.4 mmol Cu(NO_3_)_2_·3H_2_O were added in the suspension of CeO_2_. After that, the Na_2_CO_3_ aqueous solution (0.50 M) was treated as the precipitant to obtain the Cu/CeO_2_ precursor. Finally, the powder was calcined at 400 °C for 4 h in air.

### Catalytic oxidation of CO

To evaluate the activity in CO oxidation reaction, the experiments were carried out in a continuous-flow fixed-bed glass tube reactor (6.0 mm inner diameter). In a typical run, a continuous flow of the reactant mixture containing 1 vol % CO, 15 vol % O_2_, and N_2_ balance was passed through the reactor with a total flow rate of 36 mL min^−1^. A definite amount of catalyst (300 mg) was added to the isothermal region of the reactor tube. A series of Cu/CeO_2_ catalysts were tested to explore the effect of morphology, the exposed crystal plane of CeO_2_ and crystal sizes on CO oxidation reaction. The CO oxidation reaction under humid condition was also conducted by passing the N_2_ stream for adding water vapor to the carrier gas (3 vol %). After each round of reaction, the composition of the gas was detected with an online GC-7890 II gas chromatograph equipped with a thermal conductivity detector and a molecular sieve 5A column. The CO conversion rate (X_CO_) was calculated:

$${{\rm{X}}}_{{\rm{CO}}}=\frac{({[{\rm{CO}}]}_{{\rm{in}}}{\rm{vol}}.\, \% -{[{\rm{CO}}]}_{{\rm{out}}}{\rm{vol}}. \% )}{{[{\rm{CO}}]}_{{\rm{in}}}{\rm{vol}}.\, \% }\times 100$$

### Catalyst characterization

The specific surface area and the pore diameter of the Cu/CeO_2_ samples were determined by the N_2_ adsorption-desorption isotherms with a Micromeritics ASAP 2010 instrument in accordance with the BET and BJH mehod, respectively. The BET surface area was related to six measurements at relative pressures of N_2_ in the range of 0.05–1.00. Inductively Coupled Plasma Optical Emission Spectrometry (ICP-OES) was performed on Agilent 725-ES apparatus to determine the metal loadings. The morphology of these Cu/CeO_2_ samples was confirmed by Field emission scanning electron microscopy (FE-SEM, JSM-6701F) at 30 kV. Besides, the nanostructures of the samples were also characterized through a JEOL JEM-2010 transmission electron microscope operating at 200 kV, and a suspension of the Cu/CeO_2_ samples in ethanol was drop-casted onto carbon-coated copper grids and naturally dried under ambient conditions. Powder X-ray diffraction (XRD) patters was recorded on a Rigaku D/MAX-RB X-ray diffractometer with Cu Kα radiation (λ = 1.5418 Å) in the range of 10–90°. H_2_-TPR and CO-TPR measurements were performed on the chemical adsorption instrument. The reducing gas was respectively 5 vol% H_2_ and 10% CO balanced by N_2_, and a flow rate of 40 ml min^−1^ was used, and the test was carried out from room temperature to 800 °C at a heating rate of 10 °C min^−1^. Before each measurement, the sample was purged with N_2_ at 300 °C for 2 h. X-ray photoelectron spectroscopy (XPS) was performed on a VG ESCALAB 210 Electron Spectrometer with a Mg Kα(1253.6 eV) radiation, and the spectra were corrected and treated the C1s binding energy of 284.6 eV as the standard. Raman spectroscopy was performed on a RM 2000 microscope confocal Raman spectrometer with 532 nm laser (Renishaw PLC).

## Supplementary information

Supporting Information
