# Supplementary material for: Morphology effects on surface chemical properties and lattice defects of Cu/CeO2 catalysts applied for low-temperature CO oxidation
Source: Sci Rep. 2019 Aug 19;9:12056. doi: 10.1038/s41598-019-48606-2 (PMC6700188; doi:10.1038/s41598-019-48606-2)
Supplement: Supplementary file 1 — Supporting Information [file 41598_2019_48606_MOESM1_ESM.docx]

***Supporting Information for***

Morphology effects on surface chemical properties and lattice defects of Cu/CeO_2_ catalysts applied for low-temperature CO oxidation

Fang Dong,^a^ Yu Meng,^b^ Weiliang Han,^a^ Haijun Zhao,^a^ Zhicheng Tang^a *^

^a^ State Key Laboratory for Oxo Synthesis and Selective Oxidation, and National Engineering Research Center for Fine Petrochemical Intermediates, Lanzhou Institute of Chemical Physics, Chinese Academy of Sciences, Lanzhou 730000, China

^b^ Shanxi Key Laboratory of Low metamorphic Coal Clean Ytilization, School of Chemistry and Chemical Engineering, Yulin University, YuLin 719000, China

________________________

^*^Corresponding author: *National Engineering Research Center for Fine Petrochemical Intermediates, Lanzhou Institute of Chemical Physics, Chinese Academy of Sciences, Lanzhou 730000, China.* Tel: +86 931 4968083; Fax: +86 931 8277088.

E-mail address: tangzhicheng@licp.cas.cn

**The supporting information includes:**

S1 Experimental details

Fig. S1 to S15

S2 References

**S1 Experimental details**

**S1.1 Preparation of the CeO_2_ Support with the different morphology**

The CeO_2_ nanoparticles (**CeO_2_-P**) were synthesized according to previous report [1]. First, 6.0 mmol ammonium bromide (CTAB) was dissolved in 200 mL deionized water. After that, 10 mmol Ce(NO_3_)_3_·6H_2_O was added into the mixed solution. Then, 0.5 M NaOH solution was added dropwise to the above solution until pH 9 with vigorous stirring. The followed suspension was aged at 90 °C for 3 h, centrifuged, and washed with deionized water. Hereafter, the CeO_2_-P precursor dried at 110 °C for 12 h, and the dry powders were calcined at 400 °C for 4 h in air. The CeO_2_ nanospheres (**CeO_2_-S**) were synthesized by alcothermal method [2]. The 4.6 mmol Ce(NO_3_)_3_·6H_2_O was dissolved in 2 mL deionized water. Then, the mixed acetic acid (2 mL) and glycol (52 mL) were mixed to form a uniform solution under vigorous stirring. The mixed solution was transferred into a Teflon-lined autoclave at 180 °C for 200 min. After that, the precipitation were separated by centrifugation, washed with deionized water and ethanol 3 times, dried at 75 °C overnight, and calcined at 400 °C for 4 h in air. The CeO_2_ nanorod (**CeO_2_-R**) was synthesized by a hydrothermal method. 3.0 mmol Ce(NO_3_)_3_·6H_2_O and 9 M NaOH were dissolved in 60 mL deionized water. Then, the mixed solutions were transferred into a Teflon bottle, and stirred for 30 min. After that, the autoclave was heated at 100 °C for 24 h. The obtained precipitates were separated by centrifugation, washed with deionized water and ethanol several times, and dried at 60 °C overnight, and then calcined at 400 °C for 4 h in air. The flowerlike CeO_2_ microspheres (**CeO_2_-F**) were derived from the as-prepared CeOHCO_3_ microspheres according to the former literature [3]. First, the as-prepared CeOHCO_3_ microspheres products were calcined in a tube furnace under N_2_ with a flow rate of 10 mL min^-1^ at 600 °C for 6 h, and then calcined in air at 400 °C for 4 h.

**S1.2 The calculation of CO reaction rate**

The reaction rate of CO oxidation is calculated by:

(1)


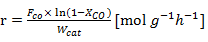


The F_co_ is CO molar flow rate, X_co_ is the conversion of CO based on CO_2_ formation, W_cat_ is the mass of catalyst in the reactor bed. When the CO conversion is below 10 %, the influence of the produced CO_2_ and H_2_O on the reaction rate can be ignored, and the composition of the reactant gas remains essentially unchanged. Therefore, lnA, xlnPco and ylnPo_2_ can be approximately considered as a constant.

**S1.3 The calculation of activation energy E_a_**

The empirical equation of the activation energy as follows:

(2)


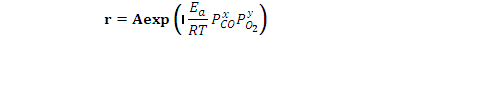

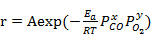


(3)


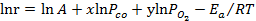


(4)


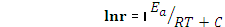

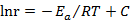


A is the preexponential factor, R is the ideal gas constant, T is the reaction temperature, Pco is the partial pressure of CO, Po_2_ is the partial pressure of O_2_, C is the constant which is equal to lnA+xlnPco+ylnPo_2_ under the CO conversion controlled below 10%.

**Table S1** Texture characteristics of the CeO_2_ supports with different morphology

| Catalysts | S_BET_*^a^* (m^2^/g) | D_p_*^a^* (nm) | V_p_*^a^* (cm^3^/g) |
| --- | --- | --- | --- |
| CeO_2_-P | 180.4 | 5.33 | 0.24 |
| CeO_2_-S | 209.8 | 4.76 | 0.25 |
| CeO_2_-R | 135.7 | 10.72 | 0.36 |
| CeO_2_-F | 54.9 | 4.80 | 0.07 |

*^a^* The BET surface area, pore volume, and pore size were determined by N_2_ physical-adsorption.


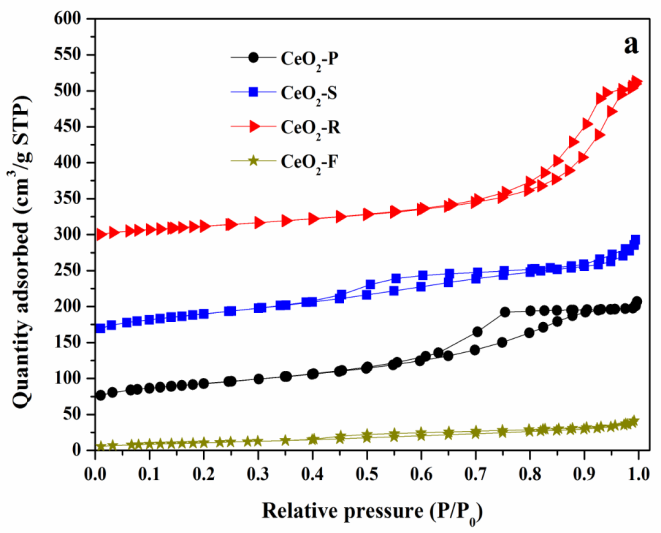

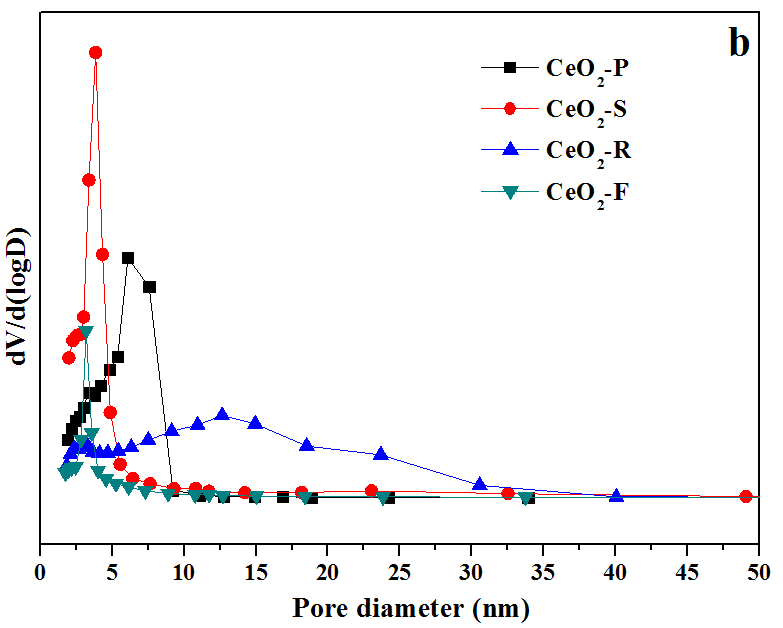

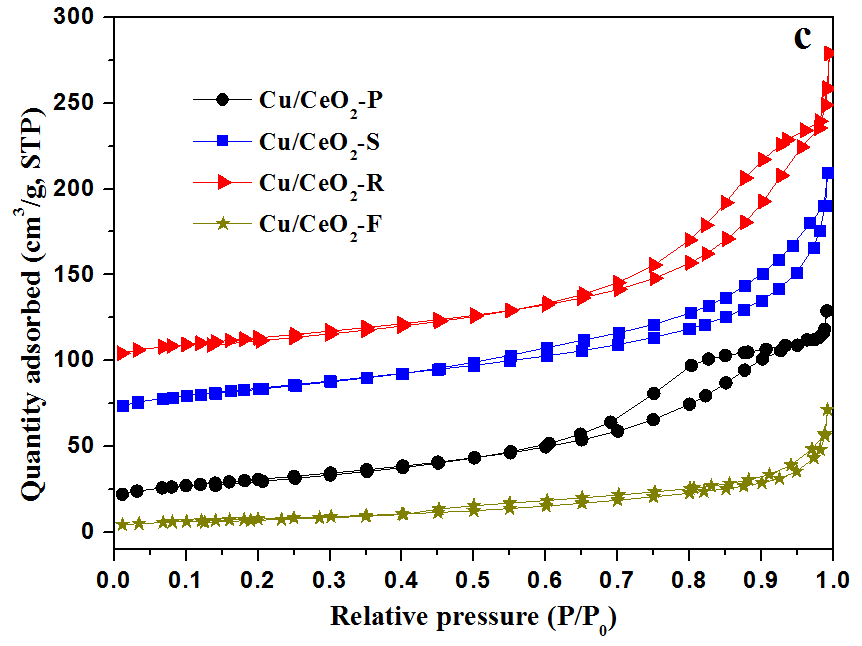

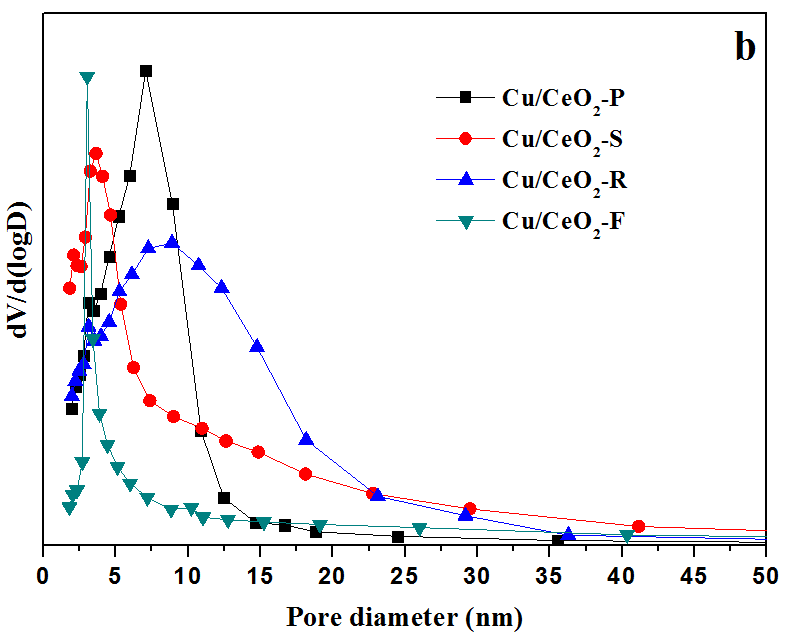


**Fig. S1** (a) Nitrogen adsorption-desorption isotherms of CeO_2_ supports, (b) pore-size distributions of CeO_2_ supports, (c) Nitrogen adsorption-desorption isotherms of the Cu/CeO_2_ catalysts, and (d) pore-size distributions of the Cu/CeO_2_ catalysts

As shown in Fig. S1a, the isotherm of CeO_2_ and Cu/CeO_2_ samples exhibited the reversible type IV isotherm, according to the IUPAC classification. The characteristic of CeO_2_ and Cu/CeO_2_ catalysts with an important contribution of mesoporous was exhibited in Fig. S1b and c, and it is concluded from the sharp increase of the pore volume at low p/p_0_. Especially, the Cu/CeO_2_-F sample appeared an inflection at p/p_0_=0.35-0.40, characteristic of capillary condensation within uniform mesopores. The very narrow hysteresis loop of Cu/CeO_2_-F catalyst reflected the absence of adsorbate pore blocking. Notably, the Cu/CeO_2_-R sample showed a type IV isotherm of a mesoporous material with a broad pore size distribution, and CuO/CeO_2_-F with a very narrow pore size distribution exhibited an isotherm typical of a low surface area material. Besides, the difference of BET specific surface area and pore volume was observed, and the reason was most probably attributable to a certain degree of pore blockage caused by the presence of copper species on the surface of CeO_2_. The dispersion of copper species closely depended on the morphology of CeO_2_ support. Totally, it is discovered that the Cu/CeO_2_-R (nanorods) with the large BET surface area might be beneficial to promote the dispersion of copper species, while the Cu/CeO_2_-F sample exhibited the smallest BET surface area, which might be disadvantage to the enhancement of copper dispersion.


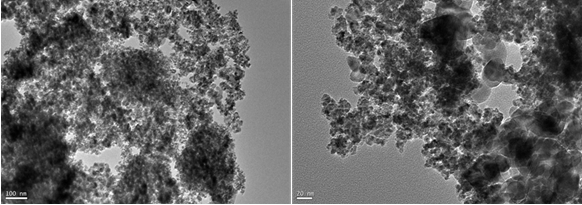


**Fig. S2** TEM images of the Cu/CeO_2_-P catalyst


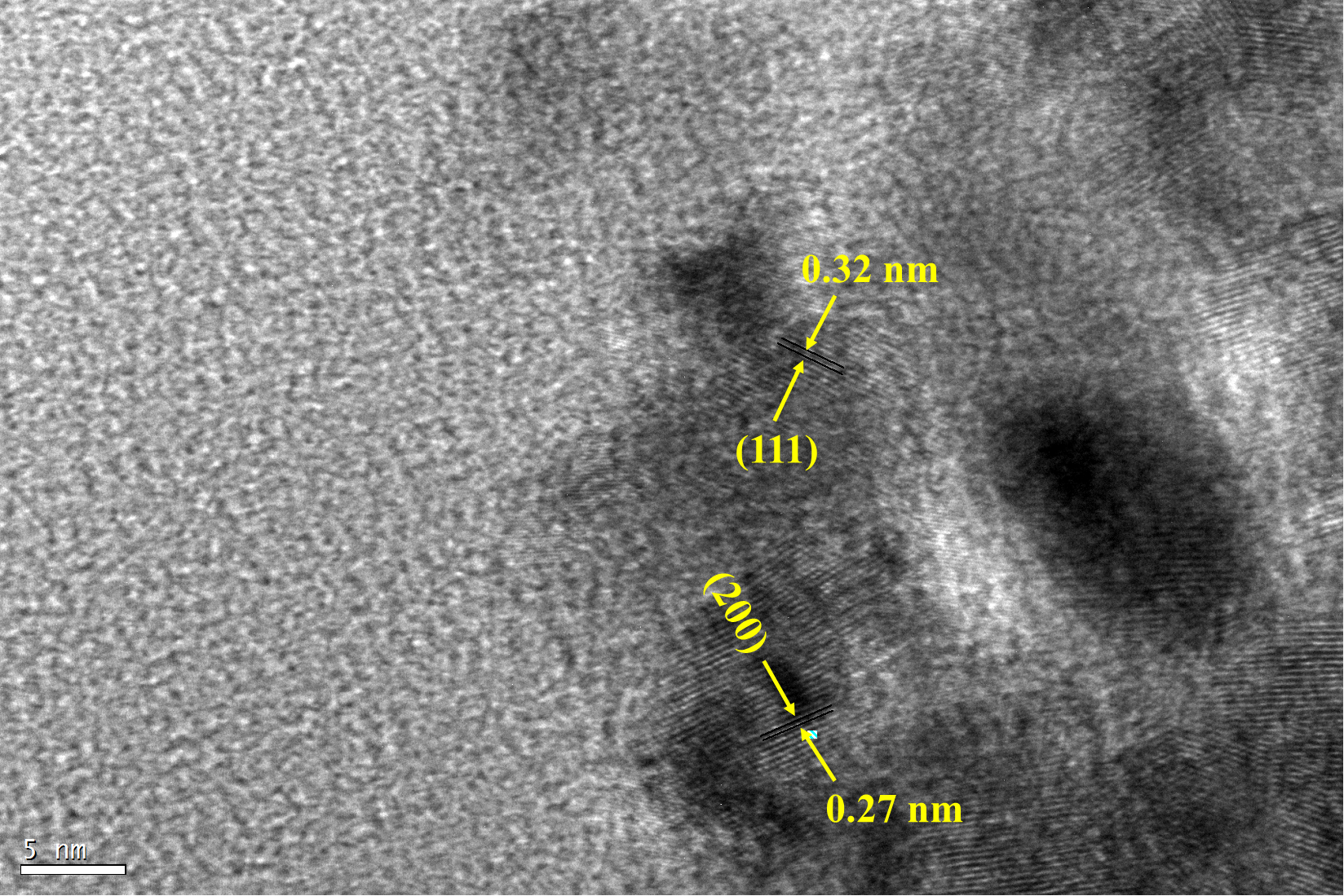

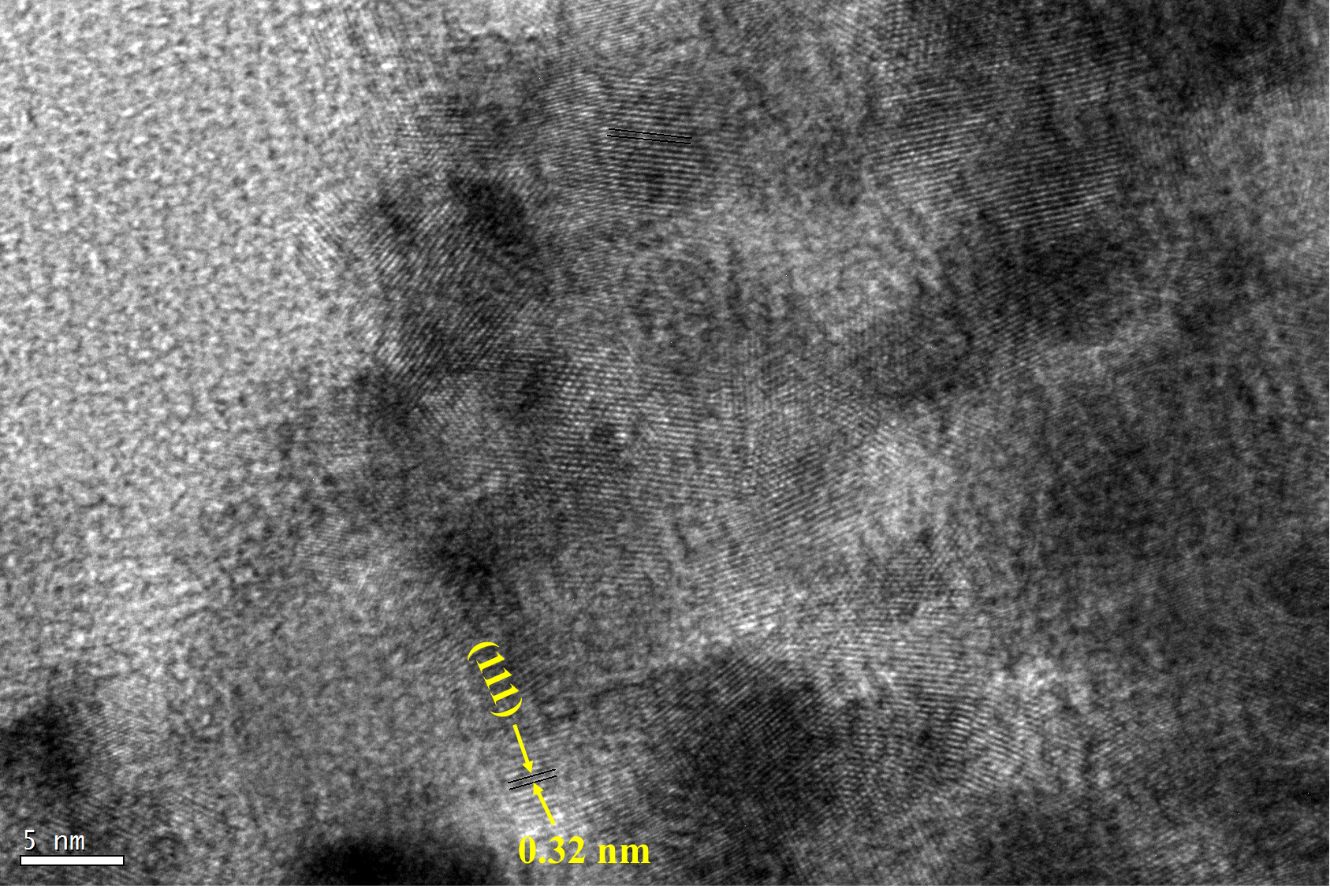


**Fig. S3** HRTEM images of the Cu/CeO_2_-P catalyst


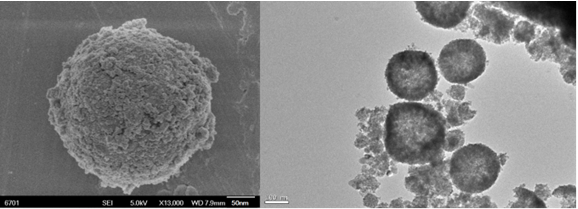


**Fig. S4** SEM and TEM images of the Cu/CeO_2_-S catalyst


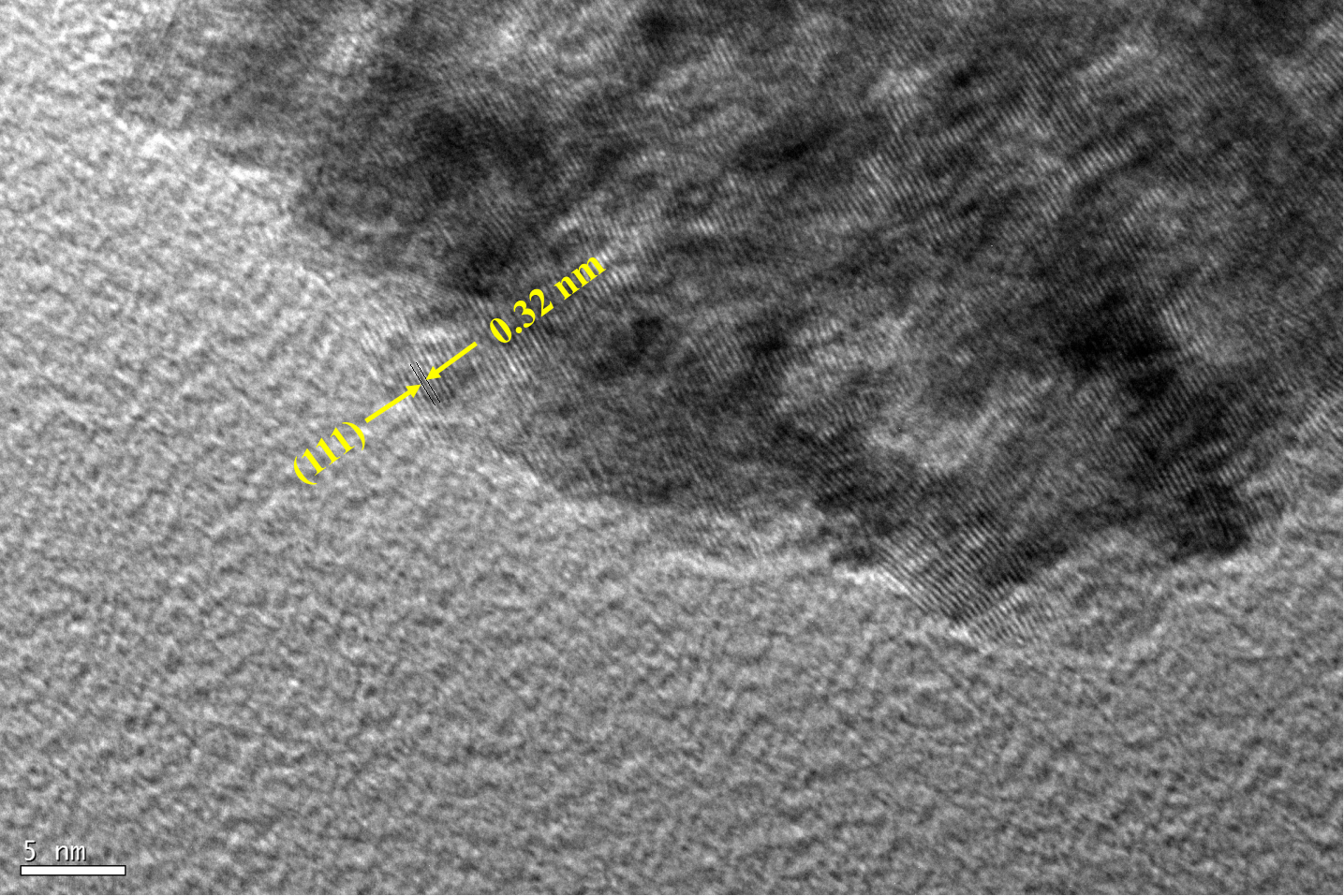

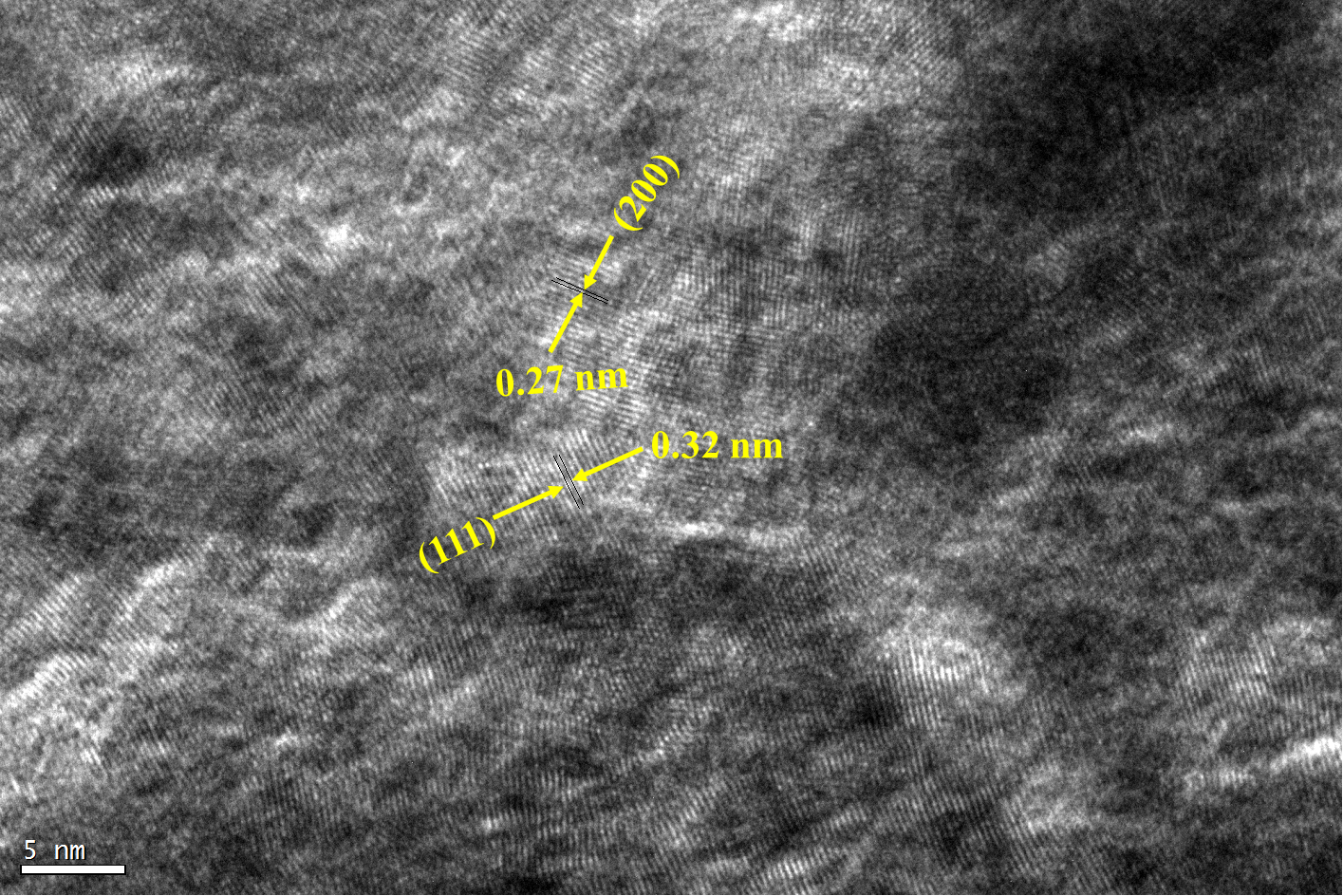


**Fig. S5** HRTEM images of the Cu/CeO_2_-S catalyst


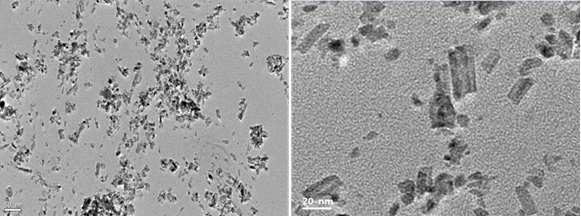


**Fig. S6** TEM images of the Cu/CeO_2_-R catalyst


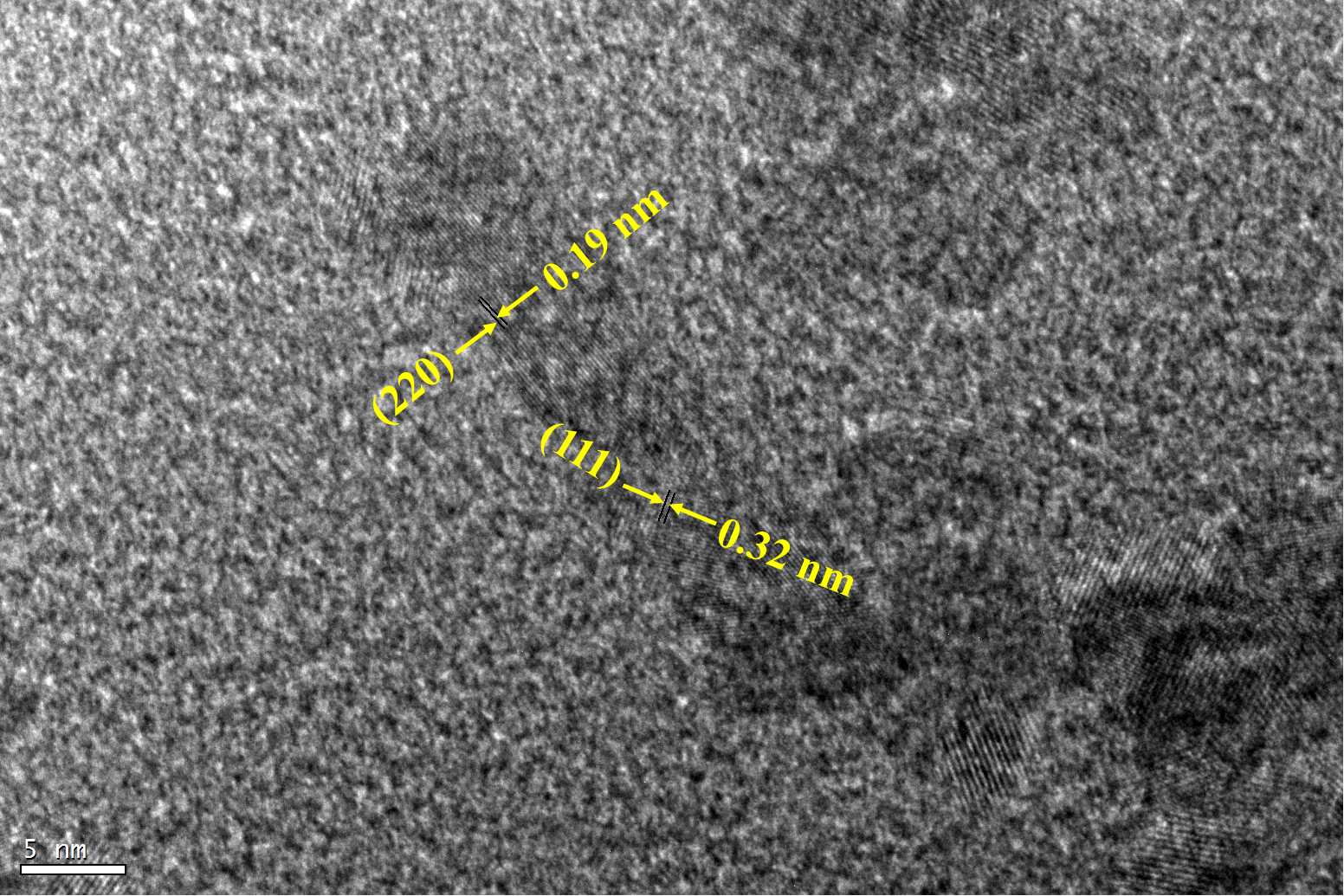

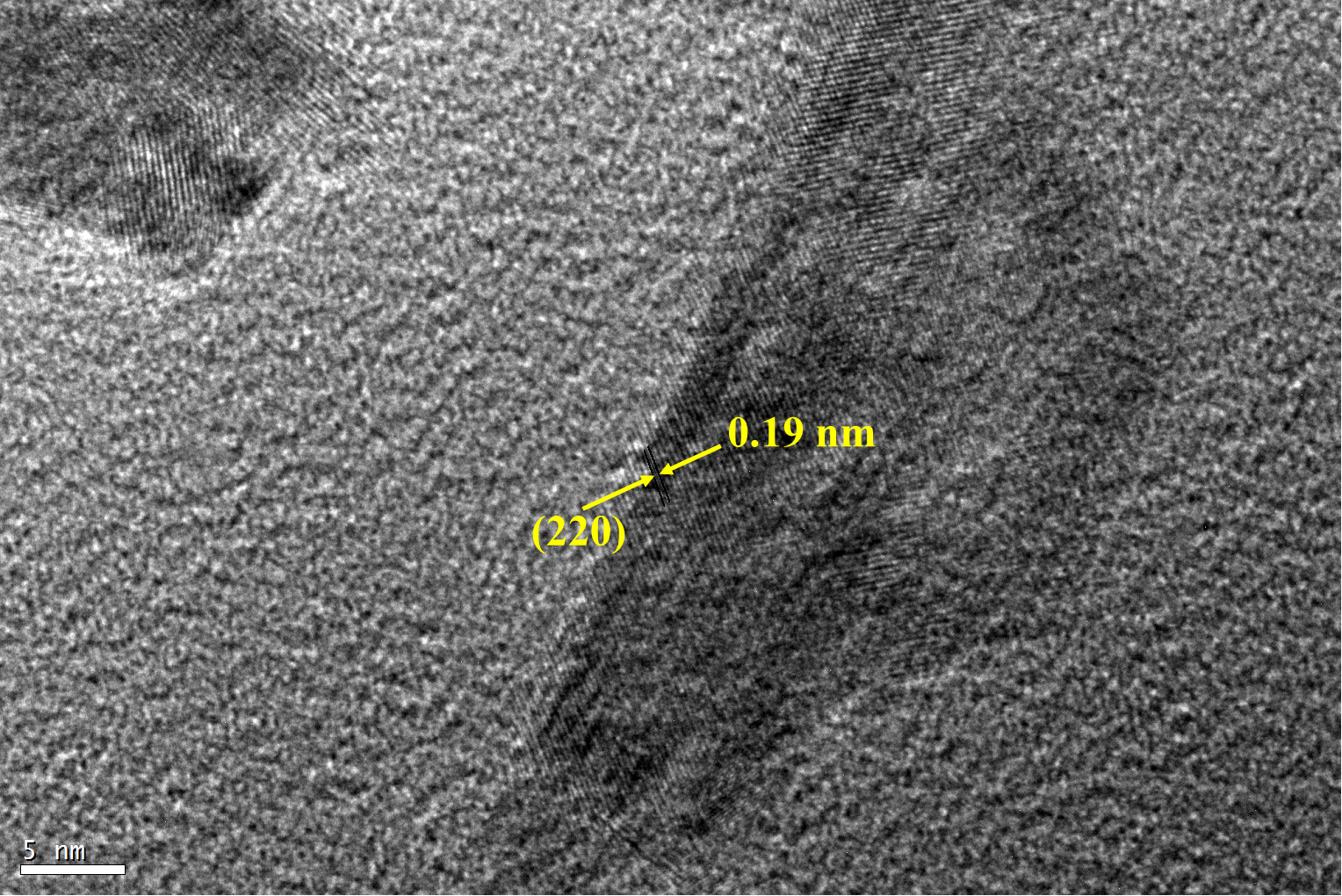

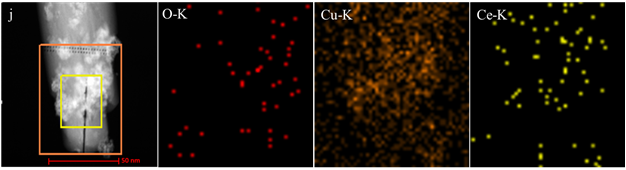


**Fig. S7** HRTEM images of the Cu/CeO_2_-R catalyst


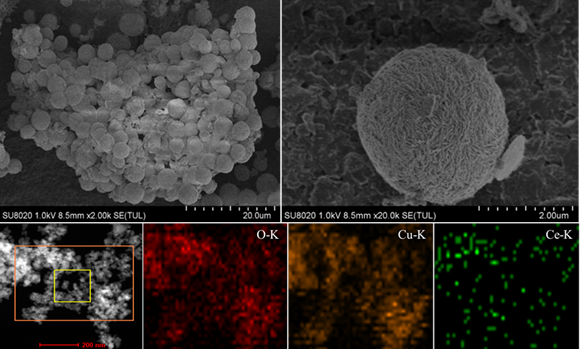


**Fig. S8** SEM images of the Cu/CeO_2_-F catalyst


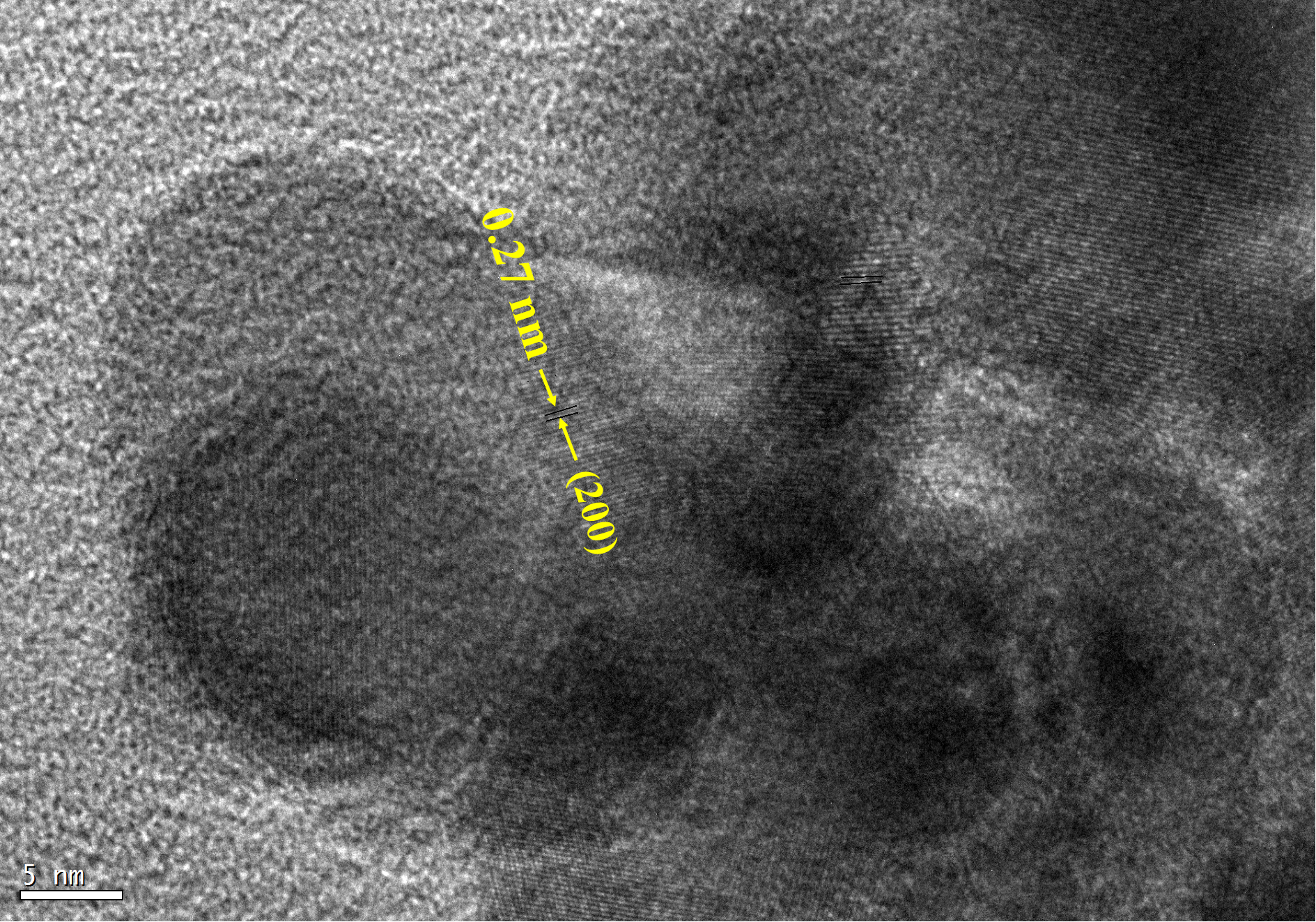

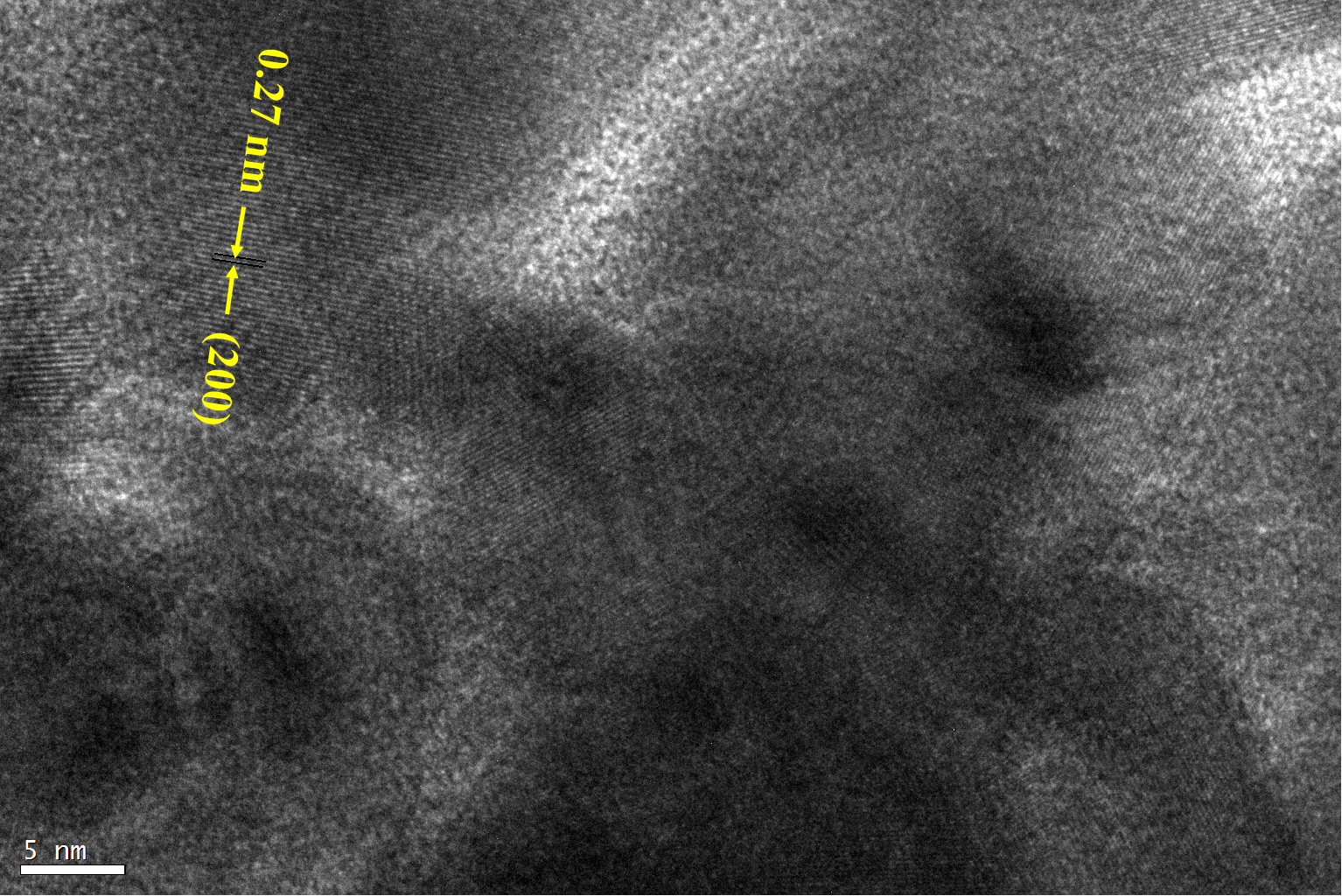


**Fig. S9** HRTEM images of the Cu/CeO_2_-F catalyst


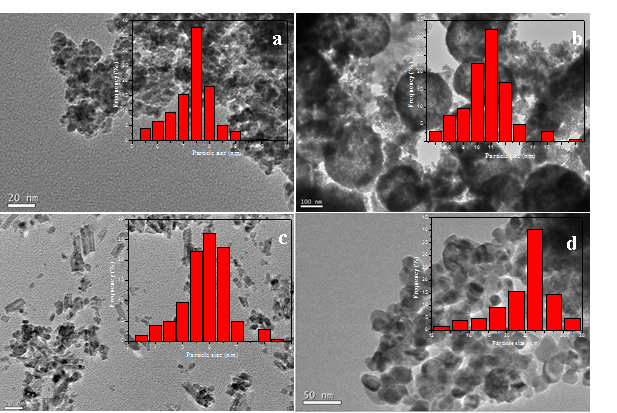


**Fig. S10** The distribution of CuO particles size over a) Cu/CeO_2_-P catalyst, b) Cu/CeO_2_-S catalyst, c) Cu/CeO_2_-R catalyst, d) Cu/CeO_2_-F catalyst from TEM analysis


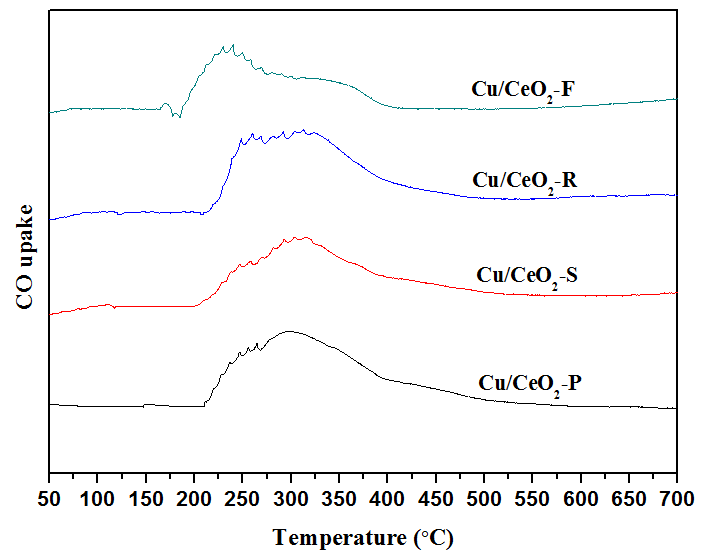


**Fig. S11** CO-TPR patterns of the Cu/CeO_2_ catalysts with different morphology

The reducibility of these Cu/CeO_2_ catalysts was also explored by CO-TPR, which used CO as reducing agent. Compared with H_2_-TPR results, CO-TPR analysis can provide more detailed information of catalyst redox properties, due to the different reducing power and chemical properties of CO and H_2_ molecular. Moreover, CO-TPR can avoid the occurrence of hydrogen overflow with respect to H_2_-TPR [4]. According to Fig. 10, it is discovered that the reduction peak of the Cu/CeO_2_-F catalyst exhibited the broad peak due to the largest copper particles size and the weak interaction of copper particles and flower-like CeO_2_ microspheres. The Cu/CeO_2_-P and Cu/CeO_2_-S catalysts showed the similar reduction peak due to the same exposed crystal planes of CeO_2_ support, which was advantage to increasing the interaction of copper species and CeO_2_ support. Notably, the Cu/CeO_2_-R catalyst exhibited more symmetrical reduction peak than other three samples because of the uniform CuO particles size. Combined with XRD and H_2_-TPR results, it was concluded that the CuO particles size played the significant role in determining the CO conversion, and the small copper particles was beneficial to promote the conversion of CO and decreased the energy barrier (E_a_) of CO oxidation reaction. Especially, the Cu/CeO_2_-F catalyst had the higher energy barrier (E_a_) of CO oxidation due to the larger copper particles size and the lower oxygen amounts.


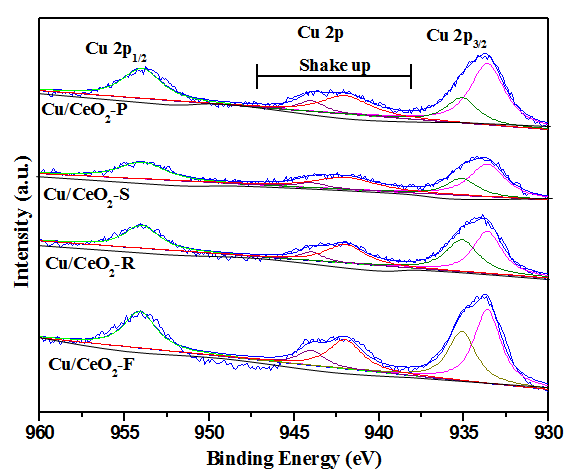


**Fig. S12** Cu 2p XPS analysis of these Cu/CeO_2_ catalysts

For Fig. S12, the XPS spectra in the Cu 2p region of the catalysts exhibited the Cu 2p_3/2_ peaks at binding energy of 933.5 eV, and the Cu 2p_1/2_ peak at binding energy of 953.5 eV [3-5]. Besides, the Cu^2+^ species was verified through the existence of shake-up peaks in the range of 936-950 eV [6]. Based on the previous literatures [5,7], the binding energy at 934.9 eV was attributed to the presence of well-dispersed Cu^2+^ species, and the lower binding energy was related to the existence of Cu^+^ species and Cu^0^ species. These four Cu/CeO_2_ catalysts all exhibited the weak shake-up peaks, indicating the existence of Cu^2+^ species on the surface of these catalysts. Compared with other three Cu/CeO_2_ catalysts, the shake-up peak of the Cu/CeO_2_-F catalyst was more obvious, suggesting that more Cu^2+^ species existed on the surface of the Cu/CeO_2_-F catalyst. Notably, the Cu 2P_3/2_ peak of the Cu/CeO_2_-F catalyst shifted toward lower binding energy than that of other three catalysts, which were very close. The above results suggested that the Cu species on the surface of the Cu/CeO_2_-P, Cu/CeO_2_-S and Cu/CeO_2_-R catalysts were similar. Besides, the surface atomic ratio of Cu/Ce was calculated through the XPS characterization in Table 3. It was discovered that the surface atomic ratio of Cu/Ce of these Cu/CeO_2_ catalyst was not proportional to the activity of CO oxidation, indicating that there had other factors to affect the performance of these Cu/CeO_2_ catalysts excluding the surface content of copper species.


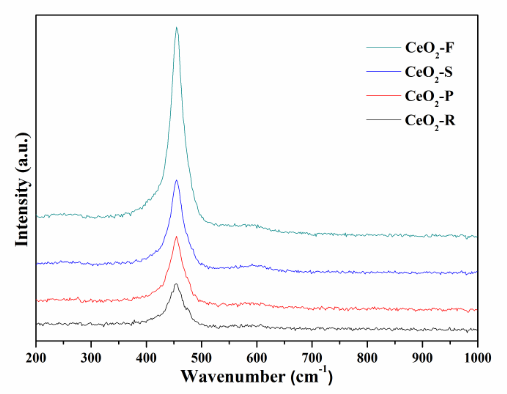


**Fig. S13** Raman spectra of CeO_2_ supports with different morphology


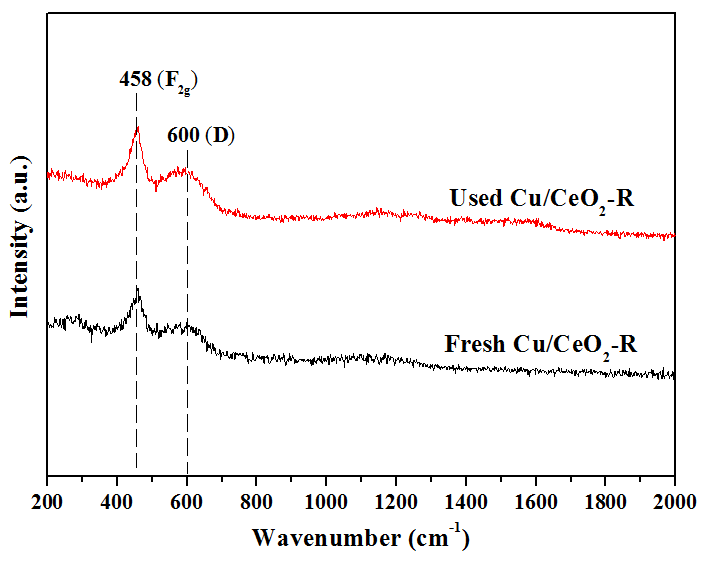


**Fig. S14** Raman spectra of Cu/CeO_2_-R catalyst before and after H_2_O resistance reaction


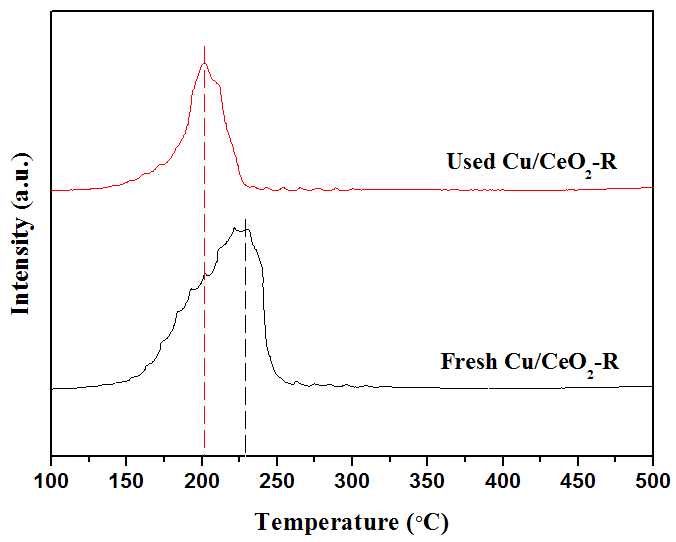


**Fig. S15** H_2_-TPR analysis of Cu/CeO_2_-R catalyst before and after H_2_O resistance reaction

For H_2_-TPR analysis, it was discovered that Cu/CeO_2_-R catalyst after H_2_O resistance reaction exhibited an asymmetric reduction peak at lower temperature than fresh Cu/CeO_2_-R catalyst, indicating the production of smaller CuO NPs, and the results was consistent with XRD results.

**S2 References**

1. Qi, L., Yu, Q., Dai, Y., Tang, C., Liu, L., Zhang, H., Gao, F., Dong, L. & Chen, Y. Influence of cerium precursors on the structure and reducibility of mesoporous CuO-CeO_2_ catalysts for CO oxidation. Appl. Catal. B: Environ. **119-120**, 308-320 (2012).
2. Liu, Z., Duchon, T., Wang, H., Grinter, D., Waluyo, I., Zhou, J., Liu, Q., Jeong, B., Crumlin, E., Matolin, V., Stacchiola, D., Rodriguez, J. & Senanayake, S. Ambient pressure XPS and IRRAS investigation of ethanol steam reforming on Ni-CeO_2_(111) catalysts: an in situ study of C-C and O-H bond scission. Phys. Chem. Chem. Phys. **18**, 16621-16628 (2016).
3. Sun, C., Sun, J., Xiao, G., Zhang, H., Qiu, X., Li, H. & Chen, L. Mesoscale Organization of Nearly Monodisperse Flowerlike Ceria Microspheres. J. Phys. Chem. B **110**, 13445-13452 (2006).
4. Wang, J., Bian, C., Tong, J., Sun, J. & Xia, S. Simultaneous Detection of Copper, Lead and Zinc on Tin Film/Gold Nanoparticles/Gold Microelectrode by Square Wave Stripping Voltammetry. Electroanal. **24**, 1783-1790 (2012).
5. Avgouropoulos, G., Ioannides, T. & Matralis, H. Influence of the preparation method on the performance of CuO-CeO_2_ catalysts for the selective oxidation of CO. Appl. Catal. B: Environ. **56**, 87-93 (2005).
6. Wen, B. & He, M. Study of the Cu-Ce synergism for NO reduction with CO in the presence of O_2_, H_2_O and SO_2_ in FCC operation. Appl. Catal. B: Environ. **37**, 75-82 (2002).
7. Avgouropoulos, G. & Ioannides, T. Selective CO oxidation over CuO-CeO_2_ catalysts prepared via the urea-nitrate combustion method. Appl. Catal. A: Gen. **244**, 155-167 (2003).
